# Apparent diffusion coefficient for genetic characterization of untreated adult gliomas: A meta-analysis stratified by methods

**DOI:** 10.1093/noajnl/vdaf103

**Published:** 2025-05-22

**Authors:** Faheem Bhatti, Joachim Strobel, Christopher Tench, Matthew Grech-Sollars, Robert A Dineen, Nico Sollmann, Stefanie Thust

**Affiliations:** Nottingham University Hospitals NHS Trust, Nottingham, UK; Radiological Sciences, Mental Health and Clinical Neuroscience, School of Medicine, University of Nottingham, Nottingham, UK; Department of Diagnostic and Interventional Radiology, University Hospital Ulm, Ulm, Germany; Department of Nuclear Medicine, University Hospital Ulm, Ulm, Germany; Division of Clinical Neurology, University of Nottingham, Nottingham, UK; Lysholm Department of Neuroradiology, National Hospital for Neurology and Neurosurgery, London, UK; Department of Computer Science, University College London, UK; NIHR Nottingham Biomedical Research Centre, Magnetic Resonance and Precision Imaging, Nottingham, UK; Sir Peter Mansfield Imaging Centre, University of Nottingham, Nottingham, UK; Children’s Brain Tumour Research Centre, University of Nottingham, Nottingham, UK; Nottingham University Hospitals NHS Trust, Nottingham, UK; Radiological Sciences, Mental Health and Clinical Neuroscience, School of Medicine, University of Nottingham, Nottingham, UK; TUM-Neuroimaging Center, Klinikum rechts der Isar, Technical University of Munich, Munich, Germany; Department of Diagnostic and Interventional Neuroradiology, School of Medicine, Klinikum Rechts der Isar, Technical University of Munich, Munich, Germany; Department of Diagnostic and Interventional Radiology, University Hospital Ulm, Ulm, Germany; Department of Brain Rehabilitation and Repair, UCL Institute of Neurology, London, UK; NIHR Nottingham Biomedical Research Centre, Magnetic Resonance and Precision Imaging, Nottingham, UK; Sir Peter Mansfield Imaging Centre, University of Nottingham, Nottingham, UK; Nottingham University Hospitals NHS Trust, Nottingham, UK; Radiological Sciences, Mental Health and Clinical Neuroscience, School of Medicine, University of Nottingham, Nottingham, UK

**Keywords:** apparent diffusion coefficient, diffusion-weighted imaging, glioma, glioblastoma, isocitrate dehydrogenase (IDH)

## Abstract

**Background:**

Isocitrate dehydrogenase (IDH) mutation and chromosome 1p19q genotyping have become fundamental to the prognostic grouping of adult diffuse gliomas. Apparent diffusion coefficient (ADC) values may enable noninvasive prediction of glioma molecular status. The purpose of this systematic review and meta-analysis was to investigate the diagnostic accuracy of ADC for IDH and 1p19q genotyping, considering measurement techniques and tumor grade.

**Methods:**

A systematic search of PubMed and Cochrane Library databases was performed in December 2024. Studies were grouped according to the ADC parameter measured and the measurement techniques used. A meta-analysis was performed, supplemented by Egger’s regression testing. The quality of studies was assessed with the QUADAS-2 tool.

**Results:**

Thirty-three studies, including a total of 4297 patients, fulfilled the inclusion criteria. IDH mutation and 1p19q deletion status were assessed by 30 and 14 studies, respectively. Pooled area under the curve (AUC) values for the prediction of an IDH mutation and 1p19q codeletion ranged from 0.743 (0.680–0.805) to 0.804 (0.689–0.919), and 0.678 (0.614–0.741) to 0.692 (0.600–0.783). No significant differences were identified between regional and volumetric measurements, between ADCmean and ADCmin values, or comparing normalized and raw ADC data.

**Conclusions:**

This meta-analysis supports ADC as an imaging biomarker in untreated gliomas, specifically to predict IDH status. ROI measurement, particularly by a single ADC_mean_, is rapid, reproducible, and appears statistically equivalent to volumetric readouts. We found no evidence for superior diagnostic accuracy by ADC normalization. Published ADC thresholds have been summarized for consideration of prospective testing across institutions.

**Key Points**

- Apparent diffusion coefficient (ADC) values support the prediction of glioma IDH status.
- Regional and volumetric ADC performance was equivalent in the meta-analysis.
- ADC thresholds are proposed for the sensitive identification of glioblastoma genetics.

**Importance of the Study**

Predicting brain tumor genotypes has become an important objective in radiological diagnosis. This is particularly the case for identifying molecular glioblastoma, which may otherwise be at risk of inequitable low-grade triage. Multiple studies have proposed apparent diffusion coefficient values as a biomarker of diffuse glioma IDH and 1p19 status. This systematic review and meta-analysis examined the entire available literature on the subject, including a variety of different measurement methods. Thresholds for prospective research and clinical trial applications are proposed.

Gliomas represent the most common primary malignancy of the central nervous system (CNS) in adults and are frequently incurable.^1–3^ Molecular markers of prognostic relevance have become fundamental in the diagnosis of gliomas as defined in the World Health Organization (WHO) 2021 Classification of CNS Tumors.^4^ Diffuse gliomas are divided into 3 genetic groups based on the presence of an isocitrate dehydrogenase gene mutation (IDH-mutant, IDH^mut^), with or without chromosome 1p19q codeletion (1p19q^codel^).^4^ Glioblastoma (GBM) is the most lethal type of glioma, characterized by the absence of an IDH mutation (IDH-wildtype, IDH^wt^) and malignant histology (WHO grade 4).^4^ In contrast, most IDH-mutant tumors are low-grade gliomas (WHO grades 2–3), divided into IDH^mut^/1p19q^retained^ astrocytomas and IDH^mut^/1p19q^codel^ oligodendrogliomas.^2,4,5^

Glioma genotyping is essential for risk stratification and to guide clinical management. GBM is treated by resection followed by radiotherapy and temozolomide chemotherapy.^6–9^ Maximizing tumor resection prolongs the survival of GBM, which creates an argument for prompt identification.^5,8,10^ A proportion of IDH^wt^ tumors display histological low-grade features but belong to the molecular class of GBM, requiring radical treatment with a risk of comparably poor outcomes.^1,2,4^ On the contrary, the survival in IDH^mut^ WHO grade 4 tumors tends to be longer than in GBM.^1^ In IDH^mut^ astrocytomas, postoperative tumor volume is independently associated with survival, whereas 1p19q^codel^ oligodendrogliomas preferentially respond to chemotherapy.^11^ A preoperative prediction of IDH status could help better utilize sequencing resources in situations where IDH sequencing is not routine for all gliomas and/or where geographical inequities contribute to diagnostic delays.^12,13^

Diffusion-weighted imaging (DWI) performed with b-values of 0 s/mm^2^ and 1000 s/mm^2^ (sometimes with an additional b-value of 500 s/mm^2^) is widely integrated into clinical glioma MRI protocols.^14,15^ From this, apparent diffusion coefficient (ADC) maps are calculated to estimate the magnitude of diffusion in each image voxel.^14^ ADC values have been negatively correlated with glioma cellularity in most studies, but other factors, including matrix composition, influence ADC.^16,17^ Several studies reported higher ADC values in IDH^mut^ gliomas compared to IDH^wt^ tumors, which may enable noninvasive prediction of glioma genotype.^18–20^ However, it is unknown whether measurement methods influence the accuracy of ADC results for glioma molecular diagnosis. Much of the published literature on the diagnostic accuracy of ADC values for characterizing gliomas predates the WHO 2021 Classification of CNS Tumors.^4^ Specifically, older studies highlighted differences in ADC parameters between high-grade and low-grade gliomas, without reporting on genetic status.^21^ This raises the possibility of a WHO grade influence on ADC diagnostic accuracy.

The purpose of this systematic review and meta-analysis was to investigate the diagnostic accuracy of ADC for glioma IDH and 1p19 status prediction by measurement techniques and considering the possible influence of glioma WHO grade.

## Methods

A literature review and meta-analysis were conducted according to the Preferred Reporting Items for Systematic Reviews and Meta-Analyses (PRISMA) guidelines.^22^ The meta-analysis component of the research was prospectively registered on July 23, 2024 with the University of Nottingham Repository (http://doi.org/10.17639/nott.7439).^23^

### Information Sources and Search Strategy

A systematic search of PubMed and Cochrane databases was commenced on September 1, 2023, and last updated on December 21, 2024, to identify studies reporting the diagnostic accuracy of ADC for glioma IDH and 1p19q status prediction. To capture the genotyping era, a filter was applied to only include studies published since 2013 (10 years prior to the commencement of the analysis). Full details of the search terms are provided in the Supplementary Material.

### Eligibility Criteria

Inclusion criteria were: original research in diffuse glioma (WHO grades 2–4), DWI/ADC or mean diffusivity/ADC calculated from diffusion tensor imaging (DTI) performed on glioma patients pretreatment, assessment of the diagnostic or prognostic value of one or more diffusion parameters for the purpose of glioma grouping (eg, WHO grade, genotype), quantitative measurements described without or alongside histogram parameters or advanced computation, and studies no more than 10 years retrospect to capture the genotyping era.^24^

Exclusion criteria were: no diffusion-weighted (DWI/ADC or diffusion-tensor imaging [DTI]) sequence interpretation, animal/laboratory measurements, studies confined to pediatric gliomas (defined as <5 adult cases), review articles, case reports of <5 cases, conference abstracts, no English full text, any previous treatment (surgery, radiotherapy, chemotherapy) and tumor types other than diffuse glioma.

### Study Selection and Data Collection Process

The titles and abstracts of all studies identified by the search were uploaded into the Rayyan online systematic review platform.^25^ Each abstract was independently screened by 2 reviewers (F.B. and J.S.). Following the unblinding of each reviewer’s screening results, conflicts were resolved through consensus. Candidate full texts were independently reviewed by the same reviewers against the inclusion and exclusion criteria, with conflicts resolved after unblinding. Each reviewer (F.B. and J.S.) extracted data from all included studies into Microsoft Excel (Microsoft Excel for Mac, Version 16.88, Microsoft Corporation). The complete data extraction was compared, and discrepancies were resolved in a consensus discussion with 2 senior authors (S.T. and N.S.).

### Data Items

Items extracted consisted of author details and publication year, study design, research purpose, patient number, age, sex, microscopic WHO grade(s) and histopathological diagnoses, immunohistochemistry methods, IDH and 1p19q status, DWI acquisition details, diffusion parameter(s) measured, measurement methods (eg, region of interest [ROI] defined as a single slice measurement(s) or volume of interest [VOI] defined as a measurement(s) obtained from multiple image slices) and interobserver testing, where published. The WHO numerical grade is Arabic throughout the manuscript in keeping with the latest WHO 2021 convention. The original grading nomenclature has been retained, where older Roman grades were used in research publications. The key outcome measure was the receiver operating characteristic (ROC) area under the curve (AUC) value of the diffusion parameter(s) used for IDH or 1p19q genotyping of gliomas.

### Study Risk of Bias Assessment

Risk of bias was assessed using the Quality Assessment of Diagnostic Accuracy Studies-2 (QUADAS-2) tool.^26^ The QUADAS-2 questions were defined through a planning consultation between the authors and are listed in Supplementary Material. Studies which did not report consecutive or random enrolments were assigned a high risk of bias in the patient selection domain. If more than one reference standard was used, a high risk of bias was assigned in the reference standard domain. For the flow and timing domain, any interval between the index and reference tests greater than 2 weeks was labelled as high risk of bias. If the interval between the index and reference tests was not specified, this was labelled as unclear risk of bias. Each study was independently assessed by 2 reviewers (F.B and J.S.) with disagreements resolved through consensus with one senior author present (N.S.).

### Statistical Analysis and Synthesis Methods

A meta-analysis was performed to estimate the diagnostic performance of ADC measurements. For studies with 95% confidence intervals (CIs) but not standard error (SE) values, the 95% CIs were used to calculate the SE values by dividing the CI range by 3.92 as specified in the Cochrane Handbook for Systematic Reviews of Interventions.^27^ For studies in which AUC values were reported without an SE value or CI, the corresponding authors were emailed 3 times to supply the missing data. If SE and/or CI values remained unconfirmed, an estimate of the SE value was calculated using Equation II described by Cortes and colleagues.^28^

Studies were grouped according to the ADC parameter measured (eg, minimum ADC [ADCmin] or mean ADC [ADCmean]), and the method of measurement (ROI or VOI). Studies which marked ROIs in the visually perceived lowest regions of the ADC map were coded as ADCmin. Studies which described placing an ROI across the largest axial tumor cross-section were classified as ADCmean. Studies taking an average ADC measurement from multiple ROIs placed within a tumor, but not targeting the lowest regions of the ADC map, were also classified as ADCmean. Studies, which targeted the visually perceived lowest ADC regions, were classed as ADCmin, even where these lowest ADC values were averaged. This grouping served the purpose of meta-analysis to align methods as closely as possible, irrespective of individual publication nomenclature. The data were further grouped based on whether absolute or normalized (eg, to contralateral normal-appearing white matter [CNAWM]) ADC measurements were recorded.

The meta-analysis was performed using the “Meta-analysis” function on JASP software (JASP Team 2024, version 0.18.3 [Apple Silicon]). The fixed effects model was initially used with Cochran’s Q statistic to test for residual heterogeneity, whereby a threshold of *P* < .05 indicated significant residual heterogeneity. In the case of significant residual heterogeneity, the maximum likelihood model was applied instead. Results are displayed in forest plots with summary estimates presented. Funnel plots were produced, and ‘Egger’s regression test’ was performed to assess for plot asymmetry if at least 5 studies were available for analysis. To analyze the influence of WHO grade on AUC values, a linear regression was performed using the quoted AUC values and proportion of WHO grade 4 tumors in each cohort where at least 5 studies reported a specific ADC parameter. Linear regression was performed using GraphPad Prizm Cloud (GraphPad Software, www.graphpad.com).

## Results

### Study Selection and Overview

**Figure 1.**
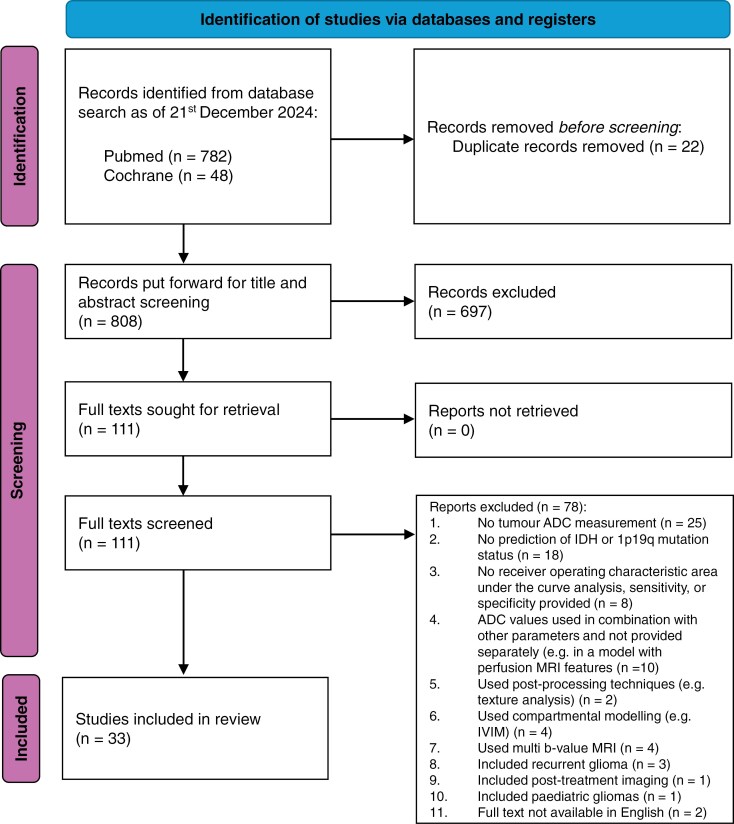
PRISMA flow diagram.

The database searches yielded 808 unique studies, of which 33 were eligible for inclusion in this review. A PRISMA flow diagram summarizing the study selection process is provided in Figure 1.

Thirty-three studies, including a total of 4297 patients, were included in the final analysis, with a mean of 130 patients per study and a range of 11 to 475. All 33 studies were retrospective. Across the 33 studies, 30 studies assessed prediction of IDH mutation status,^29–58^ and 14 studies assessed prediction of 1p19q codeletion status.^47–57,59–61^Tables 1–3 summarize the data extracted from each of the 33 included studies grouped according to whether they assessed IDH mutation, both IDH mutation and 1p19q codeletion, or 1p19q codeletion alone.

**Table 1.** Summary of Studies Assessing IDH mutation Only

Study	Research purpose	WHO grades	Cohort size and composition	Age (years), Sex	ROI or VOI methods	Results
Villanueva-Meyer JE et al. 2018	To identify MRI markers predictive of IDH mutational status in grade II diffuse gliomas (DGs) and evaluate the complementary roles of MRI features and IDH mutational status to better predict outcomes for these patients.	2	100 WHO 2. (IDH-wt = 22, IDH-mut = 65)	IDH-wt Median 58. IDH-mut Median 41. Sex NS	VOI Tumor delineated on all axial slices to calculate min, mean, and max ADC of tumor. Necrosis, cysts, hemorrhage, vessels avoided	- ADCmin: AUC 0.905 (0.830–0.954), Cutoff <0.9 × 10^–3^, Sens 91%, Spec 76%, *P* < .001.
Thust SC et al. 2021	To compare volumetric and regional ADC measurement techniques for glioma genotyping with a focus on IDH status prediction.	2,3	283 (WHO 2 and 3) (IDH-wt = 79, IDH-mut 1p19q-retained = 104, IDH-mut 1p19q-codel = 100)	Median 30, IQR 33–53. 164M, 119F	ROI and VOI Regional ADC measurements: 3, small, 30–40 mm^2^ ROIs placed in visually perceived lowest ADC portions of glioma, remaining in solid component. ADCmean: 1 large ROI placed on the largest axial tumor cross-section. Calcium, cysts, hemorrhage, vessels were avoided from ROIs. VOI: whole tumor segmentation incorporating entire T2-weighted signal abnormality.	- VOI ADCmin: Cutoff 0.81 × 10^–3^ mm^2^/s, Sens 68.4%, Spec 60.3%, AUC 0.68 (0.61–0.75). -VOI rADCmin (fifth percentile): Cutoff 1.08, Sens 68.4%, Spec 61.3%, AUC 0.72 (0.66–0.79). - VOI ADCmean: Cutoff 1.19 × 10^–3^ mm^2^/s, Sens 77.2%, Spec 64.2%, AUC 0.78 (0.72–0.84). -VOI rADCmean: Cutoff 1.60, Sens 86.8%, Spec 60.8%, AUC 0.82 (0.76–0.88). - ROI ADCmin: Cutoff 1.07 × 10^–3^ mm^2^/s, Sens 82.3%, Spec 61.3%, AUC 0.79 (0.73–0.85). -ROI rADCmin: Cutoff 1.40, Sens 85.5%, Spec 62.3%, AUC 0.81 (0.76–0.86). - ROI ADCmean: Cutoff 1.34 × 10^–3^ mm^2^/s, Sens 84.8%, Spec 60.3%, AUC 0.81 (0.75–0.86). -ROI rADCmean: Cutoff 1.75, Sens 86.8%, Spec 62.3%, AUC 0.83 (0.77–0.88). *P* < .001 for all.
Thust SC et al. 2018	To investigate if quantitative ADC measurements can predict genetic subtypes of non-gadolinium-enhancing gliomas, comparing whole tumor against single-slice analysis.	2,3	44 (WHO 2 = 26, WHO 3 = 18) (IDH-wt = 14, IDH-mut 1p19q-retained = 16, IDH-mut 1p19q-codel = 14)	IDH-wt Mean 53+/−14. IDH-mut 1p19-retained Mean 33.9+/−8.6. IDH-mut 1p19q-codel Mean 38.9+/−8.3. 22M, 22F	ROI and VOI ROI placed on largest tumor cross-section, sparing the tumor margin. VOI: whole tumor segmentation incorporating entire T2-weighted signal abnormality.	-ADCmean (VOI): Cutoff 1201(×10^–6^ mm^2^/s), sens 0.83, spec 0.86, AUC 0.85. -rADCmean (VOI): Cutoff 1.65, sens 0.80, spec 0.92, AUC 0.86. -ADCmean (ROI) first observer: Cutoff 1.83, sens 0.86, spec 1.00, AUC 0.93. -ADCmean (ROI) second observer: Cutoff 1.76, sens 0.86, spec 0.91, AUC 0.88.
Xiong J et al. 2016	To assess whether DTI metrics could aid the noninvasive detection of IDH mutations and their correlations with tumor proliferation and microvascular density (MVD) in oligodendroglial tumors.	2,3	90 (WHO 2 = 54, WHO 3 = 36). (IDH-mut = 67, IDH-wt = 23) (Oligodendroglioma = 29, Anaplastic oligodendroglioma = 24, Oligoastrocytoma = 25)	WHO II Mean 40+/−10. WHO III Mean 46+/−11 42M, 38F	ROI 4–6 ROIs placed in a solid tumor. The lowest ADC from the ROIs drawn by 2 observers was averaged and used as the minimum ADC value. ROIs were also placed in peritumoral region to calculate peritumoral ADC. Calcification, cysts, hemorrhage, necrosis avoided.	-ADCmin: Cutoff 0.81, Sens 78.7%, Spec 79.2%, AUC 0.77. -rADCmin: Cutoff 1.19, Sens 80.9%, Spec 76.9%, AUC 0.80.
Maynard J et al. 2020	To evaluate clinically available MRI parameters for predicting IDH status in patients with glioma.	2,3	339 (Study sample = 290, test sample = 49) (WHO 2 and 3) (Study sample: IDH-wt = 82, IDH-mut 1p19q-retained = 107, IDH-mut 1p19q-codel = 101) (Test sample: IDH-wt = 9, IDH-mut 1p19q-retained = 21, IDH-mut 1p19q-codel = 19)	Study sample: Median 10, IQR 33–52, Range 17–77. 169M,121F. Test sample age and sex NS.	ROI 1) 3 ROIs (30–40 mm^2^) placed in visually perceived lowest ADC portions of each tumor; the lowest ROI mean ADC measurement is designated as ADCmin. 2) One large ROI placed to cover the largest axial tumor cross-section; used as ADCmean. Tumor margins, necrosis, hemorrhage, calcification avoided.	-rADCmean: AUC 0.83. -ADCmin: AUC 0.78. -rADCmin: AUC 0.8. -ADCmean: AUC 0.81.
Wasserman JK et al. 2015	To determine whether pathological and/or radiological variables exist that can reliably distinguish IDH1-R132H-positive from IDH1-R132H-negative tumors and to identify variables associated with early mortality.	3	37 WHO 3. (Anaplastic astrocytoma = 28: IDH R132H-mut = 12, IDH-wt = 16. Anaplastic oligoastrocytoma = 9: IDH R132H-mut = 6, IDH-wt = 3)	Mean 68, Range 20–81. 16M, 21F	ROI Small ROI (25 mm^2^) placed in region of lowest apparent ADC, by visual inspection, to determine ADCmin values.	- ADCmin: Cutoff 0.950 × 10^–3^ mm^2^/s, Sens 76.9%, Spec 65.2%, AUC 0.711 (0.534–0.887), *P* = .033.
Su CQ et al. 2019	To examine whether texture analysis of DWI combined with conventional MRI could non-invasively predict IDH1 mutational status in anaplastic gliomas.	3	52 WHO 3. (IDH-mut *n* = 21: 11 anaplastic astrocytoma, 10 anaplastic oligodendroglioma. IDH-wt *n* = 25 (13 anaplastic astrocytoma, 12 anaplastic oligodendroglioma)	Mean = 47.8+/−12, Range 18–72 25M, 21F	VOI Tumor manually outlined on contrast-enhanced T1WI as areas of abnormal enhancement and non-enhancing tumor. Vessels, necrosis, and edema avoided.	- ADCentropy, AUC 0.724 (0.572–0.845), Cutoff >5.763, Sens 71.4%, Spec 76%.
Du N et al. 2022	To explore the correlation between MRI morphological characteristics, ADC parameters and pathological grade and IDH gene phenotypes of gliomas.	1,2,3,4	166 (WHO 1 = 12, WHO 2 = 31, WHO 3 = 18, WHO 4 = 105) (IDH-wt = 112, IDH-mut = 48) *No IDH status for 6 patients.	Mean 51.1+/−15.9, Range 14–85. 92M, 74F	ROI 1) ADCmin: 3 different 20–30 mm^2^ ROIs placed on visually determined lowest ADC; mean taken as ADCmin. 2) ADCmean: ROI plotted as large as possible on largest transverse cross-section of tumor. Cysts, calcification, necrosis, vessels avoided.	-ADCmin: AUC 0.653 (0.561–0.745), Cutoff 0.98, Sens 45.83, Spec 83.04. -ADCmean: AUC 0.643 (0.555–0.731), Cutoff 1.05, Sens 75.00, Spec 58.04. -rADCmin: AUC 0.656 (0.566–0.746), Cutoff 1.14, Sens 62.50, Spec 66.96. -rADCmean: AUC 0.652 (0.562–0.742), Cutoff 1.40, Sens 70.83, Spec 59.82.
Gihr G et al. 2022	To investigate (I) the potential of ADC histogram analysis for distinguishing LGGs and HGGs and (II) whether those parameters are associated with Ki-67 immunolabelling, the IDH1 mutation profile and the MGMT promoter methylation profile.	1,2,3,4	82 (WHO 1 = 7, WHO 2 = 19, WHO 3 = 11, WHO 4 = 45) (IDH-wt = 58, IDH-mut = 19) *No IDH status for 5 patients.	WHO I + II Mean 34. WHO III + IV Mean 62. 34M, 48F	VOI Tumor volumes were manually drawn in T1W or T2W images along the border of visible signal alteration (contrast-enhancing region or T2W hyperintense region) in every slice of detectable tumor. Volume used for histogram analysis.	- ADC Entropy, AUC 0.8040 (0.6849–0.9231), *P* < .0001. Cutoff < 5.488, Sens 0.73. Spec 0.97. - ADCmax, AUC 0.7314 (0.6054–0.8573), *P* = .0026. - Skewness, AUC 0.7486 (0.6235–0.8737), *P* = .0012.
Springer E et al. 2022	To use MR Fingerprinting-derived T1 and T2 relaxation maps to differentiate diffuse gliomas according to IDH mutation.	2,3,4	24 (WHO 2 = 10, WHO 3 = 5, WHO 4 = 9) (WHO2Diffuse Astrocytoma = 7: IDH-mut = 6, IDH-wt = 1. WHO 2Oligodendroglioma IDH-mut 1p19q-codel = 3. WHO III Anaplastic astrocytoma = 4: IDH-mut = 3, IDH-wt = 1. WHO III anaplastic oligodendroglioma IDH-mut 1p19q-codel = 1. WHO IV GBM n = 9: IDH-mut = 1, IDH-wt = 8.)	Mean 58.6, Range 23–77. 15M, 9F	ROI ROIs marked on 1) solid part of tumor with and without contrast enhancement (mean number of ROIs per case = 2.7), 2) perilesional NAWM (less than or equal to 1cm from tumor or peritumoral edema), 3) perilesional NAWM less than or equal to 1 cm distant from the tumor or from peritumoral edema, and 4) contralateral frontal lobe NAWM. Necrosis and hemorrhage are avoided.	- ADCmean: AUC 0.875, *P* < .001.
Liu S et al. 2022	To explore the feasibility of DWI metrics to predict the histologic subtypes and genetic status of gliomas noninvasively.	2,3,4	111 (WHO 2 = 36, WHO 3 = 32, WHO 4 = 43) (IDH-wt = 65, IDH-mut = 45) *No IDH status for 1 patient.	Mean 44.3+/−12.1. 58M, 53F	ROI 4 ROIs manually placed within solid components of tumors coregistered on T2WI. Cysts, necrosis, haemorrhage, calcification avoided.	-- ADCmean: AUC 0.777 (0.688,0.865), cutoff 0.0012, sens 88.4, spec 67.7. - rADCmean: AUC 0.836 (0.757,0.914), cutoff 1.60, sens 82.2, spec 80.0.
Kamble AN et al. 2023	Hypothesize that glioma can be stratified into 3 types using a flow chart of 4 yes/no questions, which correlate with the 3 glioma types in the 2021 WHO classification. Propose that radiological stratification would have prognostic implications if correlated with the WHO classification.	2,3,4	475 (Training set = 275, Validation set = 200) (WHO 2, 3, and 4) (Training set: IDH-wt = 124, IDH-mut 1p19q-retained = 54, IDH-mut 1p19q-codel = 21) (Validation set: IDH-wt = 106, IDH-mut 1p19q-retained = 48, IDH-mut 1p19q-codel = 46)	Training set: Type I Mean 47, Type II Mean 45, Type III Mean 55. 152M, 122F. Validation set: Type I Mean 45, Type II Mean 38, Type III Mean 56. 107M, 93F.	ROI ROI was placed as homogenously as possible to calculate average tumor ADC after excluding tumor necrosis. Necrosis excluded.	- ADCmean Training dataset: Cutoff 1.12, sens 82.1, spec 74.2, AUC 0.841, *P* <.0 < .0001. - ADCmean Validation dataset: Cutoff 1.20, sens 72.9, spec 64.9, AUC 0.748, *P* < .0001.
Xie Y et al. 2021	To compare the efficacy of parameters from multiple diffusion magnetic resonance imaging for the prediction of IDH1 genotype and assessment of cell proliferation in gliomas.	2,3,4	91 (WHO 2 = 27, WHO 3 = 20, WHO 4 = 43) (IDH-wt = 49, IDH-mut = 41)	IDH-mut Median 53, IQR 46.5–58. IDH-wt Median 41, IQR 34.75–50.25. 48M, 43F	ROI 3–6 ROIs manually placed in solid part of tumor parenchyma (defined as contrast-enhancing areas on T1WI, if no enhancement then the area of abnormal signal on T2FLAIR and T2FSE). The minimum ADC from each ROI was used to calculate tumor ADC values. Calcification, cysts, hemorrhage, edema, and necrosis were excluded.	WHO II and III tumors: -ADCmin: AUC 0.751, Sens 59.38, Spec 93.33, Cutoff 1.084. WHO IV tumors: -No significant difference found between diffusion imaging parameters.
Zhang H et al. 2024	To assess the diagnostic utility of clinical magnetic resonance spectroscopy and diffusion-weighted imaging in distinguishing between histological grading and isocitrate dehydrogenase (IDH) classification in adult diffuse gliomas.	2,3,4	247 (WHO 2 = 76, WHO 3 = 66, WHO 4 = 105) (IDH-wt = 125, IDH-mut = 122)“	Mean 46.96+/−13.92, Range 19–85 141M, 106F	ROI ROI placed on largest axial tumor slice. Mean ADC recorded. Calcification, cysts, hemorrhage, necrosis, and vessels are avoided.	- ADCmean: Cutoff < 9.22 × 10^2^ mm^2^/s, Sens 77.8%, Spec 78.0%, AUC 0.81, *P* < .001.
Cindil E et al. 2022	Evaluate the diagnostic performance of DWI MRI parameters in the non-invasive prediction of IDH mutation status in HGGs.	3,4	56 (WHO 3 and 4) (GBM IDH-wt = 25, GBM IDH-mut = 10, Anaplastic astrocytoma IDH-wt = 10, Anaplastic astrocytoma IDH-mut = 13)	IDH-mut Mean = 49+/−17. IDH-wt Mean = 58+/−14. 31M, 27F	ROI 1–3 ROIs manually placed on darkest areas on the tumor core that corresponded to the enhancing tumor. Lowest ROI ADC value used. Calcium, cysts, hemorrhage, vessels avoided.	-ADCmin: AUC 0.686 (0.795–0.950), Cutoff 0.954, Sens 0.74, Spec 0.66, PPV 0.77, NPV 0.58, Accuracy 0.68.
Lee S et al. 2015	To explore the difference between isocitrate dehydrogenase (IDH)-1/2 gene mutation-positive and -negative high-grade gliomas (HGGs) using histogram analysis of ADC maps.	3,4	52 (WHO 3 = 15, WHO 4 = 37). (WHO III Anaplastic astrocytoma = 15: IDH-mut = 9, IDH-wt = 6. WHO IV GBM = 37: IDH-mut = 7, IDH-wt = 30)	Mean 49.81+/−14.5, Range 22–72. 32M, 20F	VOI Tumor borders were manually drawn in each section of co-registered T2WI. ADC histogram parameters generated.	- ADCmean, AUC 0.707 (0.564–0.825), Sens 50, Spec 91.7, cutoff > 1333.42 (×10^–6^ mm^2^/s), *P* = .0178. - ADC 10%, AUC 0.707 (0.564–0.825), Sens 50, Spec 97.2, cutoff > 797 (×10^–6^ mm^2^/s), *P* = .0250. - ADC 50%, AUC 0.690 (0.547–0.825), Sens 43.7, Spec 91.7, cutoff > 1299 (×10^–6^ mm^2^/s), *P* = .0256.
Halefoglu AM et al. 2023	To investigate whether MRI features can determine IDH mutation in HGG.	4	170 WHO 4. (IDH-wt GBM = 146, IDH-mut astrocytoma = 24).	Mean 57.81+/−12.01. 103M, 67F	ROI 3 ROIs of similar size placed on visually perceived darkest regions of ADC map. Mean of 3 ROIs used as ADCmin. Method of ADCmean is unclear. Cysts, calcification, hemorrhage, necrosis avoided.	- ADCmean: Cutoff ≤ 0.879 × 10^–3^ mm^2^/s, Sens 83.65%, Spec 76.19%, PPV 94.60%, NPV 48.50%, AUC 0.866 (0.770–0.963), *P* < .01. - ADCmin: Cutoff ≤ 0.765.67 × 10^–3^ mm^2^/s, Sens 77.88%, Spec 80.95%, PPV 95.30%, NPV 42.50%, AUC 0.860 (0.760–0.960), *P* < .01. - rADCmin: Cutoff ≤ 1.002 × 10^–3^ mm^2^/s, Sens 91.35%, Spec 85.71%, PPV 96.90%, NPV 66.70%, AUC 0.939 (0.886–0.992), *P* < .01.
Uetani H et al. 2023	To investigate the most useful clinical and MRI parameters for differentiating IDH mut and wt glioblastomas.	4	327 WHO 4. (IDH wt = 306, IDH mut = 21)	Mean 65, Range 24–89 194M, 133F	ROI 4 or more circular ROIs placed within solid tumor, targeting regions with relatively low ADC. Necrosis, hemorrhage, and vessels avoided.	- ADCmean Reader 1: Cutoff ≥ 1.014, Sens 55.0%, Spec 70.3%, Acc 69.3%, AUC 0.548 (0.383–0.712). - ADCmean Reader 2: Cutoff ≥ 0.976, Sens 85.0%, Spec 40.7%, Acc 43.5, AUC 0.61 (0.486–0.734). - ADCmin Reader 1: Cutoff ≥ 1.014, Sens 45.0%, Spec 74.7%, Acc 72.8%, AUC 0.533 (0.364–0.701). - ADCmin Reader 2: Cutoff ≥ 0.866, Sens 75.0%, Spec 45.9%, Acc 47.7%, AUC 0.539 (0.412–0.665).
Xing Z et al. 2019	To evaluate the contribution of DWI in the enhancing and peri-enhancing region for discriminating IDH genotypes, and the diagnostic values of combining 2 techniques in the peri-enhancing region compared with those in the enhancing region.	4	75 WHO 4. (IDH-wt = 65, IDH-mut = 10)	IDH-mut Mean = 40.70+/−10.77. IDH-wt Mean = 52.23+/−12.71. 41M, 34F	ROI At least 5 non overlapping ROIs placed in the solid enhancing portion of tumor; mean value of the ROI of the lowest ADC value was used as ADCmin-tumor (ADCmin-t). 5 ROIs placed in the peri-tumoral, non-enhancing region; mean value of the ROI of the lowest ADC value was used as ADCmin-peritumoral region (ADCmin-p). Necrosis, cysts, haemorrhage, vessels avoided.	-rADCmin: AUC 0.703, cutoff 0.98, Sens 90%, Spec 55.93%, PPV 25.7%, NPV 97.10%.

Summary of study author, year of publication, main research purpose, composition of study cohort (WHO grade, IDH status, 1p19q status, sex), methods of ADC measurements, and key results for studies assessing IDH mutation only. Studies are listed from low to higher WHO grade(s).

Abbreviations: ADC, Apparent Diffusion Coefficient; AUC, Area under the curve; cMRI, Conventional MRI; CNAWM, Contralateral normal appearing white matter; CNS, Central nervous system; Codel, Codeletion; DG, Diffuse gliomas; DTI, Diffusion tensor imaging; DWI, Diffusion weighted imaging; F, Female; GBM, Glioblastoma multiforme; HGGs, High grade gliomas; IDH, Isocitrate dehydrogenase; IQR, Interquartile range; LGGs, Low grade gliomas; M, Male MRI, Magnetic Resonance Imaging; Mut, Mutant; NAWM, Normal appearing white matter; NS, Not stated; ROI, Region of interest; Sens, Sensitivity; Spec, Specificity; T1w, T1-weighted imaging. T2w, T2-weighted imaging; VOI, Volume of interest; Wt, Wild-type.

**Table 2.** Summary of Studies Assessing IDH Mutation and 1p19q Codeletion

Study	Research purpose	WHO grades	Cohort size and composition	Age, Sex	ROI or VOI methods	Results
Xiong J et al. 2016	To explore the correlations of cMRI and DTI values with the 1p/19 codeletion and IDH mutations in oligodendroglial tumors.	2,3	84 (WHO 2 = 50, WHO 3 = 34) (IDH-mut = 67, IDH-wt = 17, 1p19q-codel = 60, 1p19q-retained = 24)	Mean 41.5, Range 24–60. 40M, 44F	ROI. 4–6 ROIs placed in solid tumor. The lowest ADC from the ROIs drawn by the 2 observers was averaged and used as the as the minimum ADC value. Calcification, cysts, necrosis, hemorrhage avoided.	IDH - ADCmin: cutoff 0.85, sens 77.8%, spec 81.2%, PPV 94.2%, NPV 48.0%, AUC 0.82, *P* = .001. - rADCmin: cutoff 1.19, sens 79.4%, spec 81.2%, PPV 94.3%, NPV 50.0% AUC 0.83, *P* = .002. 1p19q - ADCmin: cutoff 1.13, AUC 0.63, Sens 62.3%, Spec 70.0%, PPV 83.7%, NPV 42.9%, *P* = .315.
Aliotta E et al. 2020	To develop an ADC analysis-based approach that can automatically identify IDHmut-noncodel LGG	2,3	227 (WHO 2 and 3, breakdown not provided.) (Internal set = 134: IDH-wt = 31, IDH-mut 1p19q-codel = 54, IDH-mut 1p19q-retained = 49.) (TCIA set = 93: IDH-wt = 18, IDH-mut 1p19q-codel = 26, IDH-mut 1p19q-retained = 49.)	Age NS. Sex NS.	VOI. Fully automated segmentation using 3D-Unet (Internal set) and GLISTRboost (TCIA set) algorithms. Generated ADC histograms.	IDHmut, 1p19q retained vs IDHwt and IDHmut, 1p19q codel. - ADCmin (Internal dataset): Cutoff 0.8 × 10^–3^ mm^2^/s, Sens 0%, Spec 100%, AUC 0.42, *P* = .04428. - ADCmin (TCIA dataset): Cutoff 0.8 × 10^–3^ mm^2^/s, Sens 0%, Spec 100%, AUC 0.46, *P* = .002. - ADCmean (Internal dataset): Cutoff 1.37 × 10^–3^ mm^2^/s, Sens 53%, Spec 91%, AUC 0.76, *P* < .00001. - ADCmean (TCIA dataset): Cutoff 1.37 × 10^–3^ mm^2^/s, Sens 55%, Spec 89%, AUC 0.81, *P* < .00001.
Aliotta E et al. 2019	To investigate lower-grade glioma grading using a machine learning technique that estimates fractional anisotropy from accelerated diffusion MR imaging scans containing only 3 diffusion-encoding directions.	2,3	41 (WHO 2 = 26, WHO 3 = 15) (IDH-wt = 15, IDH-mut 1p19q-retained = 14, IDH-mut 1p19q codel = 12)	Mean 45.9, Range 18–76. 24M, 17F.	VOI. Automated segmentation, including enhancing and non-enhancing tumor, with DeepMedic. Regions combined to generate whole tumor volumes. ADC histograms generated.	IDH - ADC75%: Sens 84+/−0.06, Spec 0.67+/− 0.05, AUC 0.81+/−0.03*, P* = .008. 1p19q - ADC50%: Sens 81+/−0.06, Spec 0.73+/− 0.04, AUC 0.83+/−0.03, *P* < .001.
Lee MK et al. 2020	To assess the diagnostic value of adding the ADC and CBV to the T2/FLAIR mismatch sign for differentiation of the IDH mutation or 1p/19q codeletion.	2,3	110 (WHO 2 = 45, WHO 3 = 65) (IDH-wt = 45, IDH-mut 1p19q-retained = 19, IDH-mut 1p19q codel = 46)	Mean 47.4+/−13.3. 56M, 54F	ROI ROIs drawn to encompass entire hyperintense lesion on FLAIR images and enhancing solid tumor on cases with contrast enhancement. ADC histograms generated.	IDH-mut 1p19q-retained vs IDH-wt - ADC10%, AUC 0.751 (0.617–0.886), Sens 84.2, Spec 63.6, Acc 69.8, *P* = .43. No ADC parameters could distinguish IDH-mut and IDH-wt tumors on multivariate analysis.
Liu D et al. 2020	To evaluate the diagnostic performance of ADC histogram parameters for differentiating the genetic subtypes in lower-grade diffuse gliomas and explore which segmentation method (ROI-1, the entire tumor ROI; ROI2, the tumor ROI excluding cystic and necrotic portions) performs better.	2,3	56 (WHO 2 = 37, WHO 3 = 19) (IDH-wt = 16, IDH-mut 1p19q-retained = 22, IDH-mut 1p19q codel = 18)	IDH mut: Mean 41.5+/−10.5, Range 23–66. IDH wt: Mean = 51.9+/−16.0, Range 21–73. 27M, 29F.	VOI VOI1: Entire tumor included. VOI2: Entire tumor, excluding cystic and necrotic regions. ADC histograms generated.	IDH -ADCmin VOI-1: Cutoff 560, Sens 62.5%, Spec 87.5%, AUC 0.749. -ADCmin VOI-2: Cutoff 543, Sens 62.5%, Spec 90.0%, AUC 0.831. IDH-mut 1p19q-codel vs IDH-mut 1p191-retained. -ADCmean VOI-1: Cutoff 1546.32, Sens 95.5%, Spec 55.6%, AUC 0.715. -ADCmean VOI-2: Cutoff 1387.97, Sens 81.8%, Spec 72.2%, AUC 0.758.
Hong EK et al. 2021	To evaluate the association of MRI features with the major genomic profiles and prognosis of WHO grade III gliomas compared with those of GBMs.	3	76 (WHO 3 = 76) (IDH-mut = 47, IDH-wt = 29. 1p19q-codel = 19, 1p19q-retained = 57) (Anaplastic astrocytoma = 57, Anaplastic oligodendroglioma = 19)	Mean 47.69, Range 19–68. 47M, 29F	VOI Tumor delineated on axial slices to contain high signal intensity lesions on T2WI and FLAIR, including cystic and necrotic regions. Multiplied by slice thickness and intersection gap to obtain tumor volume per section then summated to obtain total tumor volume. ADC histograms generated.	IDH: - ADCmean: Cutoff > 1.49, Acc 66.7%, Sens 66.7%, Spec 72.7%, AUC 0.67 (0.56–0.78), *P* = .008. 1p19q: -No significant associations between ADC and 1p19q on multivariable regression analysis.
Su X et al. 2023	To evaluate the value of quantitative MRI biomarkers for the identification of IDH mutation and 1p/19q codeletion in adult patients with diffuse glioma.	2,3,4	216 across test, training and validation set. (WHO 2,3, and 4. Breakdown not provided.) (IDH-wt = 127, IDH-mut 1p19q-retained = 33, IDH-mut 1p19q codel = 56)	Mean 45.59. 108M, 65F	VOI Automated segmentation with BraTumIA to include enhancing and nonenhancing tumor and necrosis then core tumors obtained with registration function in FSL. ADC histograms generated.	IDH: - ADCmean (test cohort): Cutoff > 1.630, Sens 93.8%, Spec 88.9%, AUC 0.913 (0.827–0.999). - ADC15% (test cohort): Cutoff > 1.186, Sens 93.8%, Spec 81.5%, AUC 0.888 (0.782–0.993). 1p19q codeletion amongst IDH mut gliomas: - ADCmean (training cohort): Cutoff > 1.397, Sens 100%, Spec 18.8%, AUC 0.409 (0.139–0.624). - ADC15% (training cohort): Cutoff > 1.266, Sens 97.500%, Spec 18.8%, AUC 0.440 (0.230–0.651).
Nuessle NC et al. 2021	To investigate the diagnostic performance of in vivo ADC-based stratification of integrated molecular glioma grades.	2,3,4	97 (WHO 2 = 37, WHO 3 = 28, WHO 4 = 32) (IDH-wt astrocytic = 44, IDH-mut astrocytic = 30, 1p19q-codel oligodendrogliomas = 23)	Mean 51.6+/−15.3. Sex NS.	VOI VOI manually delineated around entire tumor volume on FLAIR sequences. Necrosis, edema, and vessels avoided.	IDH: -ADCmean: AUC 0.883. 1p19q codeletion among IDH mut gliomas: -ADCmean: AUC 0.699.
Ma X et al. 2023	To investigate apparent diffusion coefficient (ADC) as imaging biomarker for preoperatively identifying glioma genotypes based on the 2021 World Health Organization (WHO) classification of CNS tumors.	2,3,4	159 (WHO 2, 3, and 4. Breakdown not provided.) (IDH-wt GBM = 81, IDH-mut 1p19q-retained astrocytoma = 46, IDH-mut 1p19q-codel oligodendroglioma = 32)	Mean 47.6+/−14.4. 93M, 66F	ROI 3 ROIs placed on visually perceived lowest regions of ADC map. Mean of ROI ADC values used as ADCmin. Calcification, cysts, haemorrhage, necrosis avoided.	IDH - rADCmin: AUC 0.86 (0.80–0.92), *P* < .0001, Cutoff 1.28, Sens 69.2%, Spec 92.6%. - ADCmin: AUC 0.84 (0.78–0.90), *P* < .0001, Cutoff 0.93 (×10^–3^ mm^2^/s), Sens 65.4%, Spec 91.4%. 1p19q codeletion among IDH-mut gliomas - rADCmin: AUC 0.67 (0.56–0.79), *P* = .009, Cutoff 1.47, Sens 52.5%, Spec 81.2%. - ADCmin: AUC 0.68 (0.57–0.80), *P* = .006, Cutoff 1.17 (×10^–3^ mm^2^/s), Sens 37.0%, Spec 100%.
Cheng Y et al. 2021	To explore the correlation between the molecular phenotypes of glioma and ADC values.	2,3,4	11 (WHO 2 = 3, WHO 3 = 4, WHO 4 = 4) (IDH-wt = 6, IDH-mut = 5. 1p19q-codel = 2, 1p19q-retained = 9.)	Age NS. Sex NS.	VOI 3D autocontouring segmentation. Vessels excluded.	IDH: - ADCmean: AUC 0.500, *P* > .05. 1p19q: - ADCmean: AUC 0.916, *P* < .05.
Cho NS et al. 2024	To compare the classification performance of normalized apparent diffusion coefficient with percentage T2-FLAIR mismatch-volume for differentiating between IDH-mutant astrocytoma and other glioma molecular subtypes.	2,3,4	104 (note 105 lesions) (WHO II = 61, WHO III = 21, WHO IV = 23) (IDH-wt = 22, IDH-mut = 83)“ *Note: only included patients with non-enhancing gliomas.	Mean 42, Range 22–79 59M, 45F	VOI nADC maps created by voxel-wise dividision of ADC by the mean ADC value of 3 spherical VOIs in the CNAWM. Tumor segmentations performed manually by one observer and refined by a semi-automated thresholding method using Analysis of Functional NeuroImages software for consistency prior to final review by a second observer. Cysts and CSF excluded.	IDH-mut astrocytoma vs IDH-mut oligodendroglioma/IDHwt gliomas - Median rADC: AUC 0.848, cutoff 1.864, Sens 70.8%, Spec 85.0%, *P* < .0001. IDH-mut astrocytoma vs IDH-mut oligodendroglioma IDH-mut astrocytoma vs IDH-mut oligodendroglioma - Median rADC: AUC 0.805, cutoff 1.864, Sens 70.8%, Spec 94.4%, *P* < .0001. IDH-mut astrocytoma vs IDH-wt glioma - Median rADC: AUC 0.883, cutoff 1.849, Sens 70.8%, Spec 95.5%, *P* < .0001.

Summary of study author, year of publication, main research purpose, composition of study cohort (WHO grade, IDH status, 1p19q status, sex), methods of ADC measurements, and key results for studies assessing both IDH mutation and 1p19 codeletion.

Abbreviations: ADC, Apparent Diffusion Coefficient; AUC, Area under the curve; cMRI, Conventional MRI; CNAWM, Contralateral normal appearing white matter; CNS, Central nervous system; Codel, Codeletion; DG, Diffuse gliomas; DTI, Diffusion tensor imaging; DWI, Diffusion weighted imaging; F, Female; GBM, Glioblastoma multiforme; HGGs, High grade gliomas; IDH, Isocitrate dehydrogenase; IQR, Interquartile range; LGGs, Low grade gliomas; M, Male; MRI, Magnetic Resonance Imaging; Mut, Mutant; NAWM, Normal appearing white matter; NS, Not stated; ROI, Region of interest; Sens, Sensitivity; Spec, Specificity; *T1w, T1 weighted imaging; T2w, T2-weighted imaging; VOI, Volume of interest; Wt, Wild-type.*

**Table 3.** Summary of Studies Assessing 1p19q Codeletion Alone

Study	Research purpose	WHO grades	Cohort size and composition	Age, Sex	ROI or VOI methods	Results
Yang X et al. 2021	To explore whether DWI can predict 1p19q codeletion status of IDH mutant LGGs.	2,3	142 (WHO 2 and 3. Breakdown not provided.) (IDH-mut 1p19q-codel = 73, IDH-mut 1p19q-retained = 69)	IDH-mut 1p19q retained: Mean 38.74+/−10.09. IDH-mut 1p19q-codel: Mean 44.94+/−10.24. 80M, 62F	ROI. At least 5 ROIs placed in solid tumor. ROI with lowest mean ADC used as ADCmin. Avoided cysts, hemorrhage, necrosis.	Identification of 1p19q codeletion in IDH mut gliomas: - nADC: Sens 76.71%, Spec 52.17%, PPV 88.30%, NPV 67.30%, AUC 0.71 (0.60–0.79), *P* < .001.
Cui Y et al. 2014	To investigate the correlation between tumor grade and prognostic biomarkers with ADC.	1,2,3,4	82 (WHO 1 = 1, WHO 2 = 35, WHO 3 = 22, WHO 4 = 24) (Oligodendroglial = 5, Oligoastrocytic = 29, Astrocytic = 48)	Mean 44.16. 42M, 40F	ROI. 4 ROIs placed in solid tumor. Mean of ROI ADC values used. Avoided CSF, cysts, necrosis, vessels.	Identification of 1p19q codeletion in 35 WHO II gliomas (32 IDHmut, 3 IDHwt): - Mean ADC: Cutoff 1565 × 10^–6^ mm^2^/s, Sens 72%, Spec 88%, AUC 0.82 (0.68–0.97), *P* = .003. - Mean nADC: Cutoff 2.0, Sens 76.5%, Spec 88%, AUC 0.81 (0.67–0.95), *P* = .004.
Latysheva A et al. 2019	To assess the value of DWI to characterize oligodendrogliomas and to distinguish them from astrocytomas.	2,3	71 (WHO 2 = 42, WHO 3 = 29) (Oligodendroglioma = 33, Astrocytoma = 38)	Mean 48+/−11.2. 35M, 36F	VOI Tumour manually delineated to include enhancing and nonenhancing regions on each axial slice. Whole tumor histogram distributions of ADC generated. Cysts avoided.	Identification of 1p19q codeletion in a cohort of astrocytomas and oligodendrogliomas: - Mean ADC: Cutoff 1094 × 10^–6^ mm^2^/s, Sens 63% (54–82), Spec 61% (51–83), PPV 65% (52; 81), NPV 73% (61; 87), AUC 0.76, *P* = .009.

Summary of study author, year of publication, main research purpose, composition of study cohort (WHO grade, IDH status, 1p19q status, sex), methods of ADC measurements, and key results for studies assessing 1p19 codeletion only.

Abbreviations: ADC, Apparent Diffusion Coefficient; AUC, Area under the curve; Codel, Codeletion; DG, Diffuse gliomas; DTI, Diffusion tensor imaging; DWI, Diffusion weighted imaging; F, Female; IDH, Isocitrate dehydrogenase; M, Male; MRI, Magnetic Resonance Imaging; Mut, Mutant; ROI, Region of interest; Sens, Sensitivity; Spec, Specificity; VOI, Volume of interest; Wt, Wild-type.

#### Cohort composition.—

The analyzed WHO grades varied across the included studies. The most common cohort mixes were WHO grade 2, 3 gliomas (*n* = 11),^30–33,47–51,59,61^ and WHO grade 2, 3, and 4 gliomas (*n* = 10).^38–41,53–58^ A breakdown of the studied WHO grades is provided in the Supplementary Material.

#### MRI field strength.—

Three studies reported using 1.5T MRI,^37,44,61^ 20 studies used 3.0T MRI,^29,32,35,36,38,39,41–43,46,47,50,51,53–57,59,60^ and 9 studies used a combination of 1.5T and 3.0T MRI from multiple vendors.^30,31,33,34,40,45,48,52,58^ The MRI field strength was not reported in one study.^49^

#### ADC measurement.—

Eighteen studies used ROI-based measurements,^32–34,36,38–42,44–47,50,55,58–60^ 13 studies used VOI-based measurements,^29,35,37,48–54,56,57,61^ and 2 studies used both ROI and VOI methods.^30,31^ Of the studies using VOI methods, 4 studies used automated tumor segmentation techniques,^48,49,53,56^ while the remaining 11 studies described manual whole tumor segmentation.^29–31,35,37,50–52,54,57,61^ For tumor segmentation, 4 studies used T2-weighted (T2w) sequences,^30,31,50,51^ one study used T2-FLAIR (FLAIR),^54^ one study used T2w and FLAIR,^52^ one study used T1-weighted (T1w) or T2w,^37^ and one used T1w alone.^35^ Three studies did not state what sequences were used for tumor segmentation.^29,57,61^ Studies were broadly consistent in excluding calcified, cystic, hemorrhagic, or necrotic regions of tumors from measurements. Of the 20 studies using ROI methods of ADC measurement, 14 studies assessed minimum ADC values (ADCmin)^30,32–34,36,41,42,44–47,55,59,60^ and 12 studies assessed mean ADC values (ADCmean).^31,33,36,38–40,44,45,49,50,58,60^ Nine studies did not describe exact ROI definitions for ADC measurements.^31,34,37,43,48–50,52,53^

Fifteen studies described measuring normalized ADC values using a comparative ROI.^29–33,36,38,39,44,46,47,55,57,59,60^ CNAWM with location not further specified (NFS) was used as a comparison in 9 studies,^29,33,36,38,39,46,55,59,60^ while contralateral normal-appearing centrum semiovale and contralateral normal-appearing posterior limb of internal capsule were listed in 4^30,31,44,57^ and 2^32,47^ studies, respectively.

Eleven studies described methods of 2 observers working in consensus to mark ROIs or VOIs.^35,36,38,39,41,44,56,57,59–61^ Thirteen studies provided data on intraclass correlation coefficient (ICC) values of ADC measurements.^29–31,33,42,45,46,50–53,55,58^ The ICC values of ADC measurements were greater than 0.80 in 12 of the 13 studies.^29–31,33,42,46,50–53,55,58^ One study reported ICC values of 0.532 and 0.598 for ROI-based ADCmean and ADCmin measurements, respectively, in a cohort of WHO 4 gliomas.^45^ In 2 studies, ADC measurements were performed by one observer.^34,50^

### Meta-analysis

Studies were grouped for meta-analysis based on the ADC parameter which they assessed. The primary groups were ADCmean (*n* = 19),^30,31,33,36,38–40,44,45,49–54,56,58,60,61^ ADCmin (*n* = 14),^29,30,32–34,36,38,41,42,44,45,47,51,55^ rADCmean (*n* = 6),^30,31,33,36,39,60^ and rADCmin (*n* = 9).^30,32,33,36,44,46,47,55,59^ Studies which provided AUC values for other ADC metrics, but not one of the 4 previously listed ADC parameters, are described separately (*n* = 4).^35,37,48,50^ The groupings of studies by ADC parameters are provided in Supplementary Material.

### IDH

#### ADCmean.—

Eight studies used ROI methods, and 7 studies used VOI methods to measure ADCmean values to classify glioma IDH mutation status. The cohorts of the 8 studies using ROI methods comprised WHO 1–4 (*n* = 1),^36^ WHO 2–3 (*n* = 2),^30,33^ WHO 2–4 (*n* = 3),^39,40,58^ and WHO 4 gliomas (*n* = 2).^44,45^ The 7 studies using VOI methods involved cohorts of WHO 2–3 (*n* = 2),^30,39^ WHO 2–4 (*n* = 4),^31,53,54,56^ and WHO 3 gliomas (*n* = 1).^52^

Two studies by Maynard et al.^33^ and Thust et al.^31^ were excluded from meta-analysis to avoid pseudoreplication, as these reported data on the same cohort of gliomas as another study in the meta-analysis.^30^ A study by Cheng and colleagues was also excluded from the meta-analysis despite specifying ADCmean values,^56^ because of retrospective fusion ROI measurements to biopsy sites, which differ from the ROI and VOI methods used in all other studies.^56^ Cheng et al. reported an AUC value of 0.500 in their cohort of 10 WHO 2–4 gliomas.^56^

**Figure 2.**
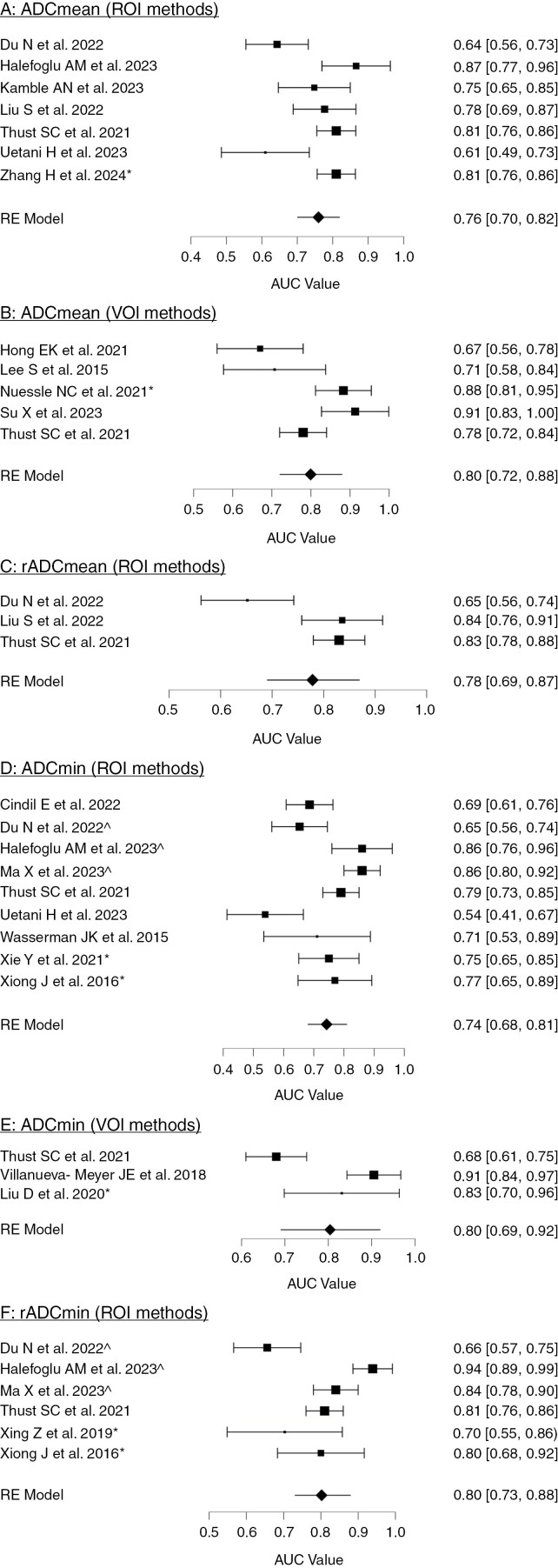
Forest plots showing the pooled estimate of AUC values from studies using each ADC parameter as a predictor of IDH mutation status. A = ADCmean (ROI methods), B = ADCmean (VOI methods), C = rADCmean (ROI methods), D = ADCmin (ROI methods), E = ADCmin (VOI methods), F = rADCmin (ROI methods). Asterix (*) denotes studies where the standard error was not provided and therefore estimated using a formula. Circumflex (^) denotes studies where 3 or more ROI measurements were averaged to determine ADC_*min*_*values.*

The pooled AUC values for ROI and VOI methods were 0.760 (0.701–0.818; I^2^ = 72.623) and 0.799 (0.719–0.879; I^2^ = 76.737), as shown in Figure 2 (Panels A and B, respectively).

#### rADCmean.—

Five studies used ROI methods, and 2 studies used VOI methods to measure rADCmean values to classify glioma IDH mutation status. The cohorts of the 5 studies using ROI methods comprised WHO 1–4 (*n* = 1),^36^ WHO 2–3 (*n* = 3),^30,31,33^ and WHO 2–4 gliomas (*n* = 1).^39^ Both studies using VOI methods analyzed cohorts of WHO 2–3 gliomas.^30,31^

Two studies were again excluded to avoid pseudoreplication.^31,33^ Due to this, 3 studies contributed to the meta-analysis of ROI methods, and no meta-analysis was possible for rADCmean studies using VOI methods. The pooled AUC value for studies using ROI methods was 0.778 (0.687–0.870; I^2^ = 79.330) as shown in Figure 2 (Panel C). Using VOI methods, Thust et al. 2021 reported an rADCmean AUC value of 0.82 (0.76–0.88) in a cohort of 283 WHO 2 and 3 gliomas.^30^

#### ADCmin.—

Eleven studies used ROI methods, and 3 studies used VOI methods to measure ADCmin values to classify IDH mutation status. In 3 of the 11 ROI studies, the generation of ADCmin measurements involved 3 or more individual ROI placements with ADC averaging.^36,44,55^ The cohorts of the studies using ROI methods comprised of WHO 1–4 (*n* = 1),^36^ WHO 2–3 (*n* = 4),^30,32,33,47^ WHO 3 (*n* = 1),^34^ WHO 2–4 (*n* = 2),^41,55^ WHO 3–4 (*n* = 1),^42^ and WHO 4 gliomas (*n* = 2).^44,45^ Of the 2 studies using VOI methods, Thust et al. assessed a cohort of WHO 2–3 gliomas, whilst Villanueva-Meyer et al. assessed a cohort of WHO 2 gliomas.^29,30^

The studies by Maynard et al.,^33^ and Xiong et al.^47^ were excluded from the ROI meta-analysis to avoid pseudoreplication, because their cohorts overlapped with those of other studies in the analysis.^30,32^ Studies using ROI methods gave a pooled AUC of 0.743 (0.680–0.805; I^2^ = 76.055; Figure 2, Panel D), whilst studies using VOI methods gave a pooled AUC of 0.804 (0.689–0.919; I^2^ = 89.969; Figure 2, Panel E).

#### rADCmin.—

Eight studies used ROI methods,^30,32,33,36,44,46,47,55^ and one study used VOI methods^30^ to measure rADCmin values to classify glioma IDH mutation status. In 3 studies, ADCmin measurements involved 3 or more individual ROI placements for averaging.^36,44,47,55^ The 8 studies using ROI methods analysed cohorts of WHO 1–4 (*n* = 1),^36^ WHO 2–3 (*n* = 5),^30,32,33,46,47^ WHO 2–4 (*n* = 1),^55^ and WHO 4 gliomas (*n* = 1).^44^ Thust et al. was the only study to use VOI rADCmin methods with a reported AUC of 0.72 (0.66–0.79).^30^

One study by Maynard et al. was again excluded to avoid pseudoreplication^33^ with another study in the meta-analysis.^30^ The study by Xiong and colleagues^47^ was also excluded to avoid pseudoreplication, as the study cohort (*n* = 84) appeared to overlap with the cohort of another study (*n* = 90) included in the meta-analysis.^51^ The pooled AUC values for the remaining 6 studies which used ROI methods, were 0.802 (0.72–0.877; I^2^ = 83.523) as shown in Figure 2 (Panel F).

### 1p19q

#### ADCmean.—

Six studies assessed ADCmean values as a classifier of glioma 1p19q codeletion status, of which 5 used VOI methods^51,53,54,56,61^ and one used ROI methods.^60^

Of the 5 studies using VOI methods, Nuessle et al.,^54^ Liu et al.,^51^ and Su et al.^53^ only assessed the prediction of 1p19q codeletion amongst IDH^mut^ gliomas while Latysheva et al. used a cohort comprising 71 WHO 2–3 gliomas, of which 33 were oligodendrogliomas and 38 were astrocytomas (note molecular status was not reported for all).^61^ Cheng et al. used a cohort of WHO 2–4 gliomas which included 6 IDH^wt^, 5 IDH^mut^/1p19q^retained^, and 2 IDH^mut^/1p19q^codel^ gliomas; however, due to their methods of retrospectively identifying ROIs from surgical biopsy sites by fusing intraoperative MRI with pre-operative imaging, this study was not included in the meta-analysis.^56^ Cheng et al. reported an AUC of 0.916 using this method.^56^ The pooled AUC of the remaining 4 studies using VOI methods was 0.692 (0.600-0.783; I^2^ = 44.948; forest plot provided in Supplementary Material).

Cui et al. were the only ones to use ROI methods in a cohort of 35 WHO 2 gliomas, of which 33 were IDH-mut, reporting an AUC of 0.820 for the prediction of 1p19q codeletion status.^60^

#### rADCmean.—

One study provided an AUC value for rADCmean as a predictor of 1p19q codeletion status. Cui et al. used ROI methods to measure rADCmean values in a cohort of 35 WHO 2 gliomas, of which 3 were IDH^wt^ and 32 were IDH^mut^, reporting an AUC value of 0.81 (0.67–0.95).^60^

#### ADCmin.—

One study assessed ADCmin values as a classifier of 1p19q codeletion status. Ma et al. reported an AUC value of 0.68 (0.57–0.80) when using the average ADC value of 3 ROIs placed on the visually perceived lowest regions of the ADC map to identify 1p19q codeletion amongst IDH^mut^ gliomas.^55^

#### rADCmin.—

Three studies measured rADCmin values using ROI methods to predict 1p19q codeletion status, giving a pooled AUC value of 0.678 (0.614–0.741; I^2^ = 0; forest plot provided in Supplementary Material).^47,55,59^ Ma et al. and Yang et al. assessed this amongst IDH-mut gliomas only while Xiong et al. used a cohort of 84 oligodendroglial tumours.^47,55,59^

### Summary of Studies Not Included in the Prior Groupings

Two studies reported ADC entropy as the best-performing ADC parameter for the classification of IDH mutation status. Su et al. used VOI methods to measure ADC entropy values in a cohort of 52 WHO 3 gliomas, obtaining an AUC of 0.724 (0.572–0.845).^35^ Gihr et al. similarly used VOI methods in a cohort of 87 WHO 1–4 gliomas and reported an AUC value of 0.804 (0.6849-0.9231).^37^

Aliotta et al. reported AUC values for 75^th^ percentile and 50^th^ percentile ADC values in the classification of IDH mutation and 1p19q codeletion status, respectively.^48^ Using automated tumor volume segmentation, in a cohort of 41 WHO 2–3 gliomas, 75th percentile ADC achieved an AUC of 0.81 (0.78–0.84) for the classification of IDH mutation status, and 50th percentile ADC achieved an AUC of 0.83 (0.80–0.86) for the classification of 1p19q codeletion.^48^

Cho et al. measured median tumor rADC values using VOI methods in a cohort of non-enhancing WHO 2–3 gliomas.^57^ They reported AUC values of 0.848 for distinguishing IDH^mut^ astrocytomas from all other gliomas, 0.805 for distinguishing between IDH^mut^ astrocytomas and IDH^mut^ oligodendrogliomas, and 0.883 for distinguishing IDH^mut^ astrocytomas from IDH^wt^ gliomas.^57^

Lee. et al assessed whether ADC values could be used to distinguish IDH^mut^,1p19q^retained^ gliomas from IDH^wt^ gliomas in a cohort of 110 WHO 2 and 3 gliomas. They reported 10th percentile ADC values, measured using ROI methods, provided an AUC of 0.751 (0.617–0.886).^50^

Aliotta et al. assessed whether IDH^mut^/1p19^retained^ gliomas could be distinguished from all other gliomas using ADC histogram parameters derived from automatically segmented tumor volumes.^49^ In a validation set of 93 WHO 2 and 3 gliomas from the Cancer Imaging Archive (TCIA) database, both ADCmean and volume of ADC > 1.5 provided an AUC of 0.81 (CIs not provided).^49^

### Influence of WHO Grade on ADC Performance for IDH Genotyping

The linear regression analysis of reported AUC values for each ADC parameter (with at least 5 observations) and the proportion of WHO 4 gliomas in the study cohorts showed no significant association between AUC and WHO grade (*P* > .05 for all ADC parameters). Full results are provided in the Supplementary Material.

### ADC Thresholds

ADC threshold values for IDH genotyping were proposed by multiple studies included in the meta-analyses, with values below the threshold denoting IDH^wt^ status in each cohort. There were 34 instances of threshold recommendations reported across the included publications, for which descriptive statistics are listed in Table 4. The maximum (Max) value specifies the threshold at which sensitivity for IDH^wt^ status would be maximized across the included studies.

**Table 4.** Proposed ADC Thresholds for IDH Genotyping According to Studies Included in the Meta-Analyses

	ROI ADCmin	ROI rADCmin	ROI ADCmean	ROI rADCmean	VOI ADCmin	VOI rADCmin	VOI ADCmean	VOI rADCmean
N studies	8	6	7	3	3	1	5	1
Median	**0.95**	**1.17**	**1.05**	**1.6**	**0.81**	**1.08**	**1.38**	**1.6**
SD	0.11	0.16	0.16	0.18	0.19	-	0.19	-
Min	0.77	0.98	0.88	1.4	0.54	-	1.19	-
Max	**1.08**	**1.40**	**1.34**	**1.75**	**0.90**	**1.08**	**1.49**	**1.6**

N, number; SD, standard deviation; Min, minimum; Max, maximum. ADC values in units of *10^–3^ mm^2^/s. rADC values have no unit.

## Risk of Bias in Studies

### Egger’s Regression Analysis for Funnel Plot Asymmetry

Funnel plots were created, and Egger’s regression analysis was performed for the ADCmean (ROI), ADCmean (VOI), ADCmin (ROI), and rADCmin (ROI) predictions of IDH mutation status, as these groups contained at least 5 studies. Funnel plots can be found in the Supplementary Material. Egger’s regression analysis revealed no significant funnel plot asymmetry for each of the ADC parameters (ADCmean [ROI] z = −1.897 [*P* = .058], ADCmean [VOI] z = −1.502 [*P* = .133], ADCmin [ROI] z = −1.449 [*P* = .147], rADCmin (ROI) z = −1.850 [*P* = .064]).

### QUADAS-2

The results of the QUADAS-2 tool risk of bias and applicability assessments, as well as information on individual studies, are provided in the Supplementary Material. All studies were retrospective. Five studies reported enrolling a consecutive or random sample of patients, whilst the remaining 28 studies were unclear regarding patient selection methods. Two studies reported using one observer to obtain ADC measurements with no consensus or comparison with a second observer. In one study, one observer obtained all ADC values with a smaller subset of cases being reviewed by a second observer. Seven studies did not specify methods of testing for IDH mutation or 1p19q deletion. Twenty-seven studies did not specify the time between the index and reference tests. Nine studies were deemed to be at high risk of bias in the flow and timing domain due to either not all patients receiving the same reference standard (eg, in some studies 1p19q co-deletion was assessed with either fluorescent immunocytochemistry or chromosomal analysis), or not all patients being included in the final analysis (eg, due to missing data on IDH mutation status). In 2 studies, the interval between the reference and index test was reported as less than 1 year, which was also allocated a high risk of bias.

## Discussion

Defining molecular status has become central to the prognostic grouping of diffuse gliomas.^4^ MRI genotyping has the potential to impact the timing and extent of tumor resection,^62^ including to accelerate radical therapy for non-contrast-enhancing glioblastoma stages,^8^ which may otherwise receive a low-grade working diagnosis.^63^ Based on numerous studies, which proposed ADC as an imaging biomarker in glioma, we investigated the performance of ADC parameters for IDH and 1p19q genotyping.

This systematic review highlights that the published literature is spread across several methods of ADC measurement. Firstly, the methods varied between the ROI and VOI approaches. The meta-analysis indicates only minor differences between the diagnostic performance of regional and volumetric values measurements (AUC ROI 0.760 vs VOI 0.799 for ADC_mean_ and ROI 0.743 vs VOI 0.804 for ADC_min_). The similarity between the diagnostic accuracy of ROI and VOI ADC for the classification of IDH mutation status has been documented previously.^30,31^ Volumetric measurements appear optimal through the capture of all representative tissue, but we found no statistical evidence for their superiority. Regional measurements are much easier to obtain and require no software beyond standard MRI viewing equipment. Thus, ROI ADC values may serve as the fastest approach with the advantage of being widely available.

For the meta-analysis, studies were grouped by measurement extent (ROI vs. VOI), by ADC parameter (mean vs. min), and according to whether ADC values were absolute or normalized, whereby each item could influence quantitative results. Surprisingly, the diagnostic performance of ADC for IDH genotyping is similar across all of these method differences. Comparing the 95% CIs of the pooled AUC values revealed no statistically significant differences between ROI and VOI measurements of ADC_mean_, rADC_mean_, ADC_min_, or rADC_min_. Drawing one ADCmean ROI across the largest glioma cross-section may be perceived as easier than deciding on the visually lowest ADC parts of a tumor. Furthermore, in several (3 of the 11) studies using ROI ADC_min_ measurements, these involved outlining 3 or more regions for averaging.

Applying the maximum threshold proposed by studies for a chosen ADC method would represent the most sensitive strategy for identifying IDH^wt^ status (at the expense of specificity over using the median). For ROI ADCmean, this would correspond to 1.3 × 10^–3^ mm^2^/s (*n* = 7 studies), or alternatively, an rADCmean threshold of 1.75 could be memorable for its similarity to perfusion thresholding, although this is based on fewer (*n* = 3) studies.^64^ ROI ADCmin values <1.1 × 10^–3^ mm^2^/s or ROI rADCmin values <1.4 should prompt suspicion for IDH^wt^ genetics.

Encouragingly, the ICC calculations exceeded 0.80 in 12 of the 13 studies which reported these, confirming high interobserver agreement for ADC.^29–31,33,42,45,46,50–53,55,58^ The only study reporting lower ICC values (0.53 for ROI ADCmean and 0.60 for ROI ADCmin) was conducted in WHO grade 4 gliomas, potentially reflecting a complexity in marking ROIs within necrotic tumors.^45^ Twenty-four of 33 studies excluded necrotic, cystic, hemorrhagic, or calcified glioma components, where present. This seems justified based on their potential to confound tumor measurements; however, little data exists on the performance of ADC according to such exclusions. In one study by Lewis et al. using ADC texture analysis, the performance for IDH typing was slightly improved by excluding necrotic gliomas.^65^

The pooled AUC estimates were lower for 1p19q genotyping than IDH genotyping, with estimates of 0.692 (0.600–0.783) for volumetric ADC mean measurements, and 0.678 (0.614–0.741) for regional rADCmin measurements. Moreover, in 8 of the 9 studies, either only IDH^mut^ gliomas were examined, or study cohorts included mostly IDH^mut^ gliomas were examined. This markedly limits the generalizability of 1p19q results for clinical practice. Glioma IDH genotyping is essential due to the strong association with survival, whereas prognosis differences are often less pronounced for IDH^mut^ subgroups, although 1p19q status may influence systemic therapy.^6^

Normalization of ADC values is aimed at reducing MRI scanner and acquisition-related variations in ADC measurements. If normalization is performed, the centrum semiovale represents a preferable target.^66^ No significant differences were observed between the pooled AUCs of normalized ADC measurements and raw ADC measurements. Several studies provided AUCs for both absolute and normalized ADC values. Du et al., Thust et al. (2018 and 2021), and Liu S et al. all reported similar AUCs when using regional measurements of ADCmean and rADCmean.^30,31,36,39^ Likewise, no significant AUC differences were identified in the 6 studies which assessed absolute and normalized regional ADCmin measurements.^30,32,36,44,47,55^

The possible effect of WHO grade distribution in the different study cohorts was explored based on the hypothesis that developing necrosis may confound ADC measurements in WHO grade 4 glioblastoma, possibly more so than in solid IDH^wt^ early disease stages.^67^ However, many studies that analyzed cohorts of different WHO grades did not report diagnostic accuracy separately for each WHO grade.^38–41,53–56,58^ The small number of studies within each meta-analysis, together with limited cohort details, precluded a further subgroup analysis. It is, however, noteworthy that one research group stratified WHO grade 2–3 gliomas by enhancement pattern (non-enhancing, solid-patchy enhancing, and rim-enhancing with necrosis) with an observation that ROI ADC values appeared strongly associated with IDH genotype in non-enhancing and solid-patchy enhancing tumors, but not in necrotic tumors.^30^

### Limitations

Our research was limited by several factors. Heterogeneity exists within the meta-analyses, with I^2^ values ranging from 0% to 89.969%. All evidence included in this review originates from retrospective research. ADC maps from 1.5T and 3T ADC MRI scanners of different vendors contributed to the analysis, with the potential to impact the quantification of absolute ADC values. Several studies omitted reporting on the blinding of observers to histopathological data, and some studies did not state the CIs of the AUC values. Studies in which the ADC values were combined with other imaging parameters (eg, visual parameters on anatomical MRI) had to be excluded if the AUC values for ADC were not provided separately.

To facilitate meta-analysis, studies were grouped according to the final consensus of 4 reviewers (F.B., J.S., NS., S.T); however, this may not reflect the entirety of methodological differences. Funnel plots could only be produced for analyses including 5 or more studies, and in a few instances, SE values were deducted by a mathematical formula.^28^ We did not explore the potential impact of manual^30^ versus automated^48,49,53,56^ glioma segmentation on ADC results, and this may represent a topic for further study where the availability of automated volumetric ADC extraction will likely grow. The future use of ADC values for glioma molecular diagnosis will most likely benefit from integration with other parameters such as visual features, physiological MRI metrics, and age.^18,33^ Furthermore, the presence of necrosis has been associated with IDHwt status and may predict this irrespective of ADC.^68^ In the future, automated segmentation may facilitate the integration of volumetric ADC measurements into clinical workflows.^69^ Furthermore, prospective research is required to validate proposed ADC thresholds.

### Practical Guidance

Whilst considering limitations of the presented data, and meta-analysis generally, it appears possible to arrive at preliminary guidance on how to perform and use ADC measurements in clinical practice. Firstly, obtaining a measurement is preferable to qualitative inspection, which is an unreliable predictor of IDH status with poor interobserver agreement.^70^ The placement of a circular ROI to measure ADCmean in the largest solid tumor cross-section (or in the largest solid tumor focus, where this is not an entire cross-section due to necrosis) is deemed suitable with a view to workflow integration. If preferred, normalized values, or alternatively ADCmin measurements, can be justified where their use is already established in local practice. We refer to Figure 1 in Maynard et al. 2020 for an example of glioma and normal white matter ROI placements, with the note that these should be interpreted as draft guidance.^33^ In support of the identification of high-risk disease, it is suggested to align measurements of ADC towards sensitive IDHwt identification, particularly for lesions of perceived “low grade” morphology. Radiological assessments should consider further factors, including age and differentials of diffuse glioma.

## Conclusion

This meta-analysis supports ADC as an imaging biomarker in untreated gliomas, specifically to predict IDH status. Published ADC thresholds have been summarized and should be considered for prospective testing. ROI measurement, particularly a single ADCmean, is rapid, reproducible, and appears statistically equivalent to volumetric readouts. We found no evidence for altered diagnostic accuracy through ADC normalization. Future research should aim to formulate numerical thresholds across multiple institutions.

## Supplementary Material

vdaf103_suppl_Supplementary_Data

vdaf103_suppl_Supplementary_Materials

vdaf103_suppl_Supplementary_Table

## Data Availability

A summary of the data extracted from all included studies is provided in the Supplementary Material together with the QUADAS-2 assessment, funnel plots and linear regression. Further information can be made available upon reasonable request. No original study data were generated in this research.
