# Supplementary material for: Apparent diffusion coefficient for genetic characterization of untreated adult gliomas: A meta-analysis stratified by methods
Source: Neurooncol Adv. 2025 May 22;7(1):vdaf103. doi: 10.1093/noajnl/vdaf103 (PMC12536491; doi:10.1093/noajnl/vdaf103)
Supplement: vdaf103_suppl_Supplementary_Materials [file vdaf103_suppl_supplementary_materials.docx]

Supplementary Material

# Search Strategy:

Search strategy in PubMed: [782 results on 21^st^ December 2024]

*https://www.ncbi.nlm.nih.gov/pubmed/?term=(((((((((((((((((brain+tumor%5BMeSH+Terms%5D)+AND+glioma%5BText+Word%5D)+OR+glioblastoma%5BText+Word%5D)+OR+oligodendroglioma)+OR+astrocytoma)+AND+%22last+11+years%22%5BPDat%5D))+AND+%22grade+%22%5BText+Word%5D)+OR+IDH%5BText+Word%5D)+OR+isocitrate%5BText+Word%5D)+OR+1p19q%5BText+Word%5D)+AND+%22last+11+years%22%5BPDat%5D))+AND+%22last+11+years%22%5BPDat%5D))+AND+imaging%5BText+Word%5D)+AND+%22last+11+years%22%5BPDat%5DAND+diffusion%5BText+Word%5D)*

Note: The search was initially performed on 1^st^ September 2023 with a filter to include studies published within the past 10 years (i.e. 2013 onwards). To ensure the review was up to date, the search was run again on 21^st^ December 2024 to identify any newly published studies. At this time, the filter was amended to include studies published within the last 11 years to ensure studies from 2013 were still captured as seen in the PubMed search above.

Search strategy in the Cochrane Library: [48 results on 21^st^ December 2024]

https://www.cochranelibrary.com/advanced-search/search-manager

#1 glioma

#2 glioblastoma

#3 oligodendroglioma

#4 astrocytoma

#5 #1 or #2 or #3 or #4

#6 "grade" or “grading” or "isocitrate dehydrogenase" or IDH or 1p19q or molecular or mutation

#7 magnetic resonance imaging

#8 diffusion

#9 #5 and #6 and #7 and #8

# QUADAS Questions

1. Did the study avoid inappropriate exclusions? (yes/no/unclear)
2. Prospective/retrospective? (prospective/retrospective/unclear)
3. Was a consecutive or random sample of patients enrolled? (yes/no/unclear)
4. Could the selection of patients have introduced bias? (high/low/unclear)"
5. Is there concern that the included patients do not match the review question? (high/low/unclear)
6. Were the index test results interpreted without knowledge of the results of the reference standard? (yes/no/unclear)
7. Two observers (independent or consensus) or only one observer? (two/one/unclear)
8. Could the conduct or interpretation of the index test have introduced bias? (high/low/unclear)
9. Is there concern that the index test, its conduct, or interpretation differ from the review question? (high/low/unclear)
10. Diagnostic reference standard used?
11. Is the reference standard likely to correctly classify the target condition? (yes/no/unclear)
12. Were the reference standard results interpreted without knowledge of the results of the index test? (yes/no/unclear)
13. Could the reference standard, its conduct, or its interpretation have introduced bias? (high/low/unclear)
14. Is there concern that the target condition as defined by the reference standard does not match the review question? (high/low/unclear)
15. The time interval and any interventions between index test(s) and reference standard?
16. Was there an appropriate interval between index test(s) and reference standard? (yes/no/unclear
17. Did all patients receive the same reference standard? (yes/no/unclear)
18. Were all patients included in the analysis? (yes/no/unclear)
19. Could the patient flow have introduced bias? (high/low/unclear)

# QUADAS-2 Summary Figures

QUADAS Risk of bias assessment of included studies.

*Proportion of included studies with low (green), high (red), or unclear (yellow) risk of bias for each of the four domains (flow and timing, reference standard, index test, and patient selection) in the QUADAS-2 assessment.*

*QUADAS Applicability assessment of included studies.*

*Proportion of included studies with low (green), high (red), or unclear (yellow) concerns regarding the three applicability domains (reference standard, index test, and patient selection) in the QUADAS-2 assessment.*

# Cohort Composition

*Table 4: Breakdown of cohort WHO grade composition*

| WHO grades in study cohort | Studies listed by Author | Total number of studies | Number of patients |
| --- | --- | --- | --- |
| 1, 2, 3, 4 | - Cui et al. 2014 - Du et al. 2022 - Gihr et al. 2022 | 3 | 330 |
| 2 | - Villanueva-Meyer et al. 2018 | 1 | 100 |
| 2, 3 | - Aliotta et al. 2020 - Aliotta et al. 2019 - Latysheva et al. 2019 - Lee M.K. et al. 2020 - Liu D. et al. 2020 - Maynard et al. 2020 - Thust et al. 2018 - Thust et al. 2021 - Xiong, Tan, Pan, et al. 2016 - Xiong, Tan, Wen, et al. 2016 - Yang et al. 2021 | 11 | 1487 |
| 2, 3, 4 | - Cheng et al. 2021 - Kamble et al. 2023 - Liu S. et al. 2022 - Ma et al. 2023 - Nuessle et al. 2021 - Springer et al. 2022 - Su X. et al. 2024 - Xie et al. 2021 - Zhang et al. 2024 - Cho et al. 2024 | 10 | 1535 |
| 3 | - Hong et al. 2021 - Su C.Q. et al. 2019 - Wasserman et al. 2015 | 3 | 165 |
| 3, 4 | - Cindil et al. 2022 - S. Lee et al. 2015 | 2 | 108 |
| 4 | - Halefoglu et al. 2023 - Uetani et al. 2023 - Xing et al. 2019 | 3 | 572 |

# Grouping of studies by ADC parameters assessed

Table 5: Breakdown of ADC parameters assessed by studies

Summary of the ADC parameters assessed by the included studies for the classification of IDH, and or 1p19q codeletion status.

| Parameter assessed | Studies assessing 1p19q only | Studies assessing both 1p19q and IDH | Studies assessing IDH only | Total number of studies using parameter |
| --- | --- | --- | --- | --- |
| ADCmean | - Cui et al. 2014 - Latysheva et al. 2019 | - Aliotta et al. 2020 - Cheng et al. 2021 - Hong et al. 2021 - Liu D. et al. 2020 - Nuessle et al. 2021 - Su X et al. 2024 | - Du et al. 2022 - Halefoglu et al. 2023 - Kamble et al. 2023 - Lee S. et al. 2015 - Liu S. et al. 2022 - Maynard et al. 2020 - Springer et al. 2022 - Thust et al. 2018 - Thust et al. 2021 - Uetani et al. 2023 - Zhang et al. 2024 | 19 |
| rADCmean | - Cui et al. 2014 |  | - Du et al. 2022 - Liu S. et al. 2022 - Maynard et al. 2020 - Thust et al. 2018 - Thust et al. 2021 | 6 |
| ADCmin |  | - Aliotta et al. 2020 - Liu D. et al. 2020 - Ma et al. 2023 - Xiong, Tan, Wen, et al. 2016 | - Cindil et al. 2022 - Du et al. 2022 - Halefoglu et al. 2023 - Maynard et al. 2020 - Thust et al. 2021 - Uetani et al. 2023 - Villanueva-Meyer et al. 2018 - Wasserman et al. 2015 - Xie et al. 2021 - Xiong, Tan, Pan, et al. 2016 | 14 |
| rADCmin | - Yang et al. 2021 | - Ma et al. 2023 - Xiong, Tan, Wen, et al. 2016 | - Du et al. 2022 - Halefoglu et al. 2023 - Maynard et al. 2020 - Thust et al. 2021 - Xing et al. 2019 - Xiong, Tan, Pan, et al. 2016 | 9 |
| Other parameters |  | - Aliotta et al. 2019 (ADC50%, ADC75%) - Lee M.K. et al. 2020 (ADC10%) - Cho et al. 2024 (median rADC). | - Gihr et al. 2022 (ADC entropy) - Su C.Q. et al. 2019 (ADC entropy) | 4 |

# Forrest plots for prediction of 1p19q codeletion

Forrest plot showing pooled estimate of AUC values from studies using **ADCmean (VOI methods)** as a predictor of 1p19q codeletion

*Asterix (*) denotes studies where standard error was not provided and therefore estimated using a formula.*


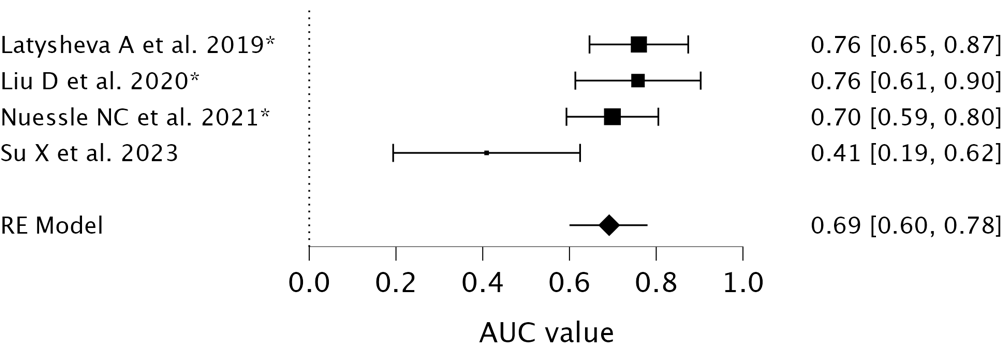


Forrest plot showing pooled estimate of AUC values from studies using **rADCmin (ROI methods)** as a predictor of 1p19q codeletion


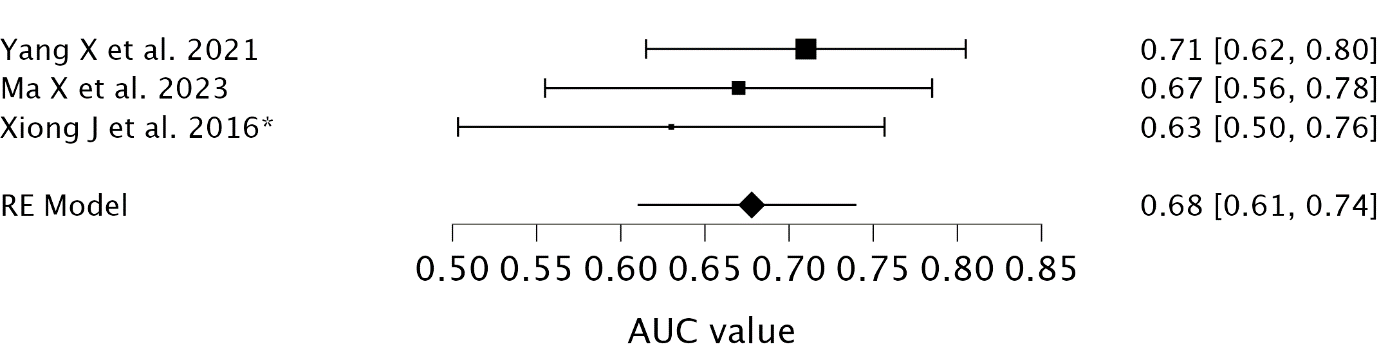


# Linear regression:

### IDH prediction by studies using ADCmean ROI measurements.


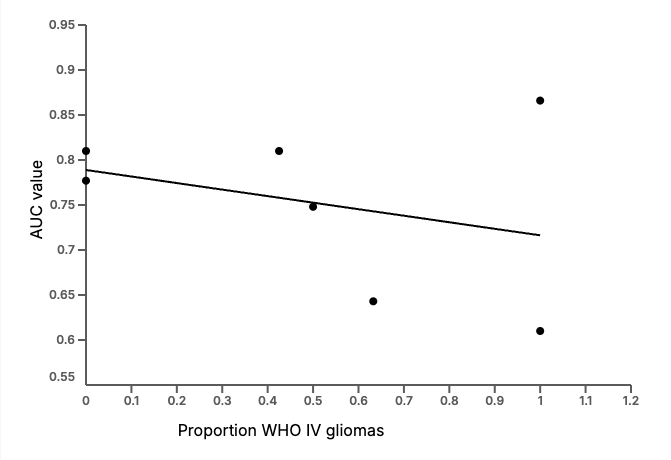


| **Best-fit values** |  |
| --- | --- |
| Slope | -0.07264 ± 0.09589 |
| Y-intercept | 0.7889 ± 0.06098 |
| X-intercept | 10.86 |
| 1/Slope | -13.77 |
| **95% Confidence Intervals** |  |
| Slope | -0.3191 to 0.1738 |
| Y-intercept | 0.6322 to 0.9457 |
| X-intercept | 2.841 to +inf |
| **Goodness of Fit** |  |
| R Square | 0.1030 |
| Sy.x | 0.09697 |
| **Is slope significantly non-zero?** |  |
| F | 0.5739 |
| DFn,DFd | 1,5 |
| P Value | 0.4829 |
| Deviation from horizontal? | Not significant |

### IDH prediction by studies using ADCmean VOI measurements.


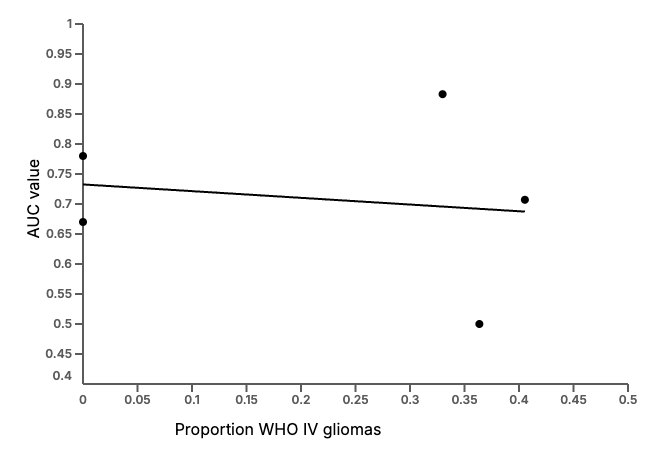


| **Best-fit values** |  |
| --- | --- |
| Slope | -0.1117 ± 0.3995 |
| Y-intercept | 0.7326 ± 0.1138 |
| X-intercept | 6.557 |
| 1/Slope | -8.951 |
| **95% Confidence Intervals** |  |
| Slope | -1.383 to 1.160 |
| Y-intercept | 0.3705 to 1.095 |
| X-intercept | 0.7014 to +inf |
| **Goodness of Fit** |  |
| R Square | 0.02541 |
| Sy.x | 0.1617 |
| **Is slope significantly non-zero?** |  |
| F | 0.07821 |
| DFn,DFd | 1,3 |
| P Value | 0.7979 |
| Deviation from horizontal? | Not significant |

### IDH prediction by studies using ADCmin ROI measurements.


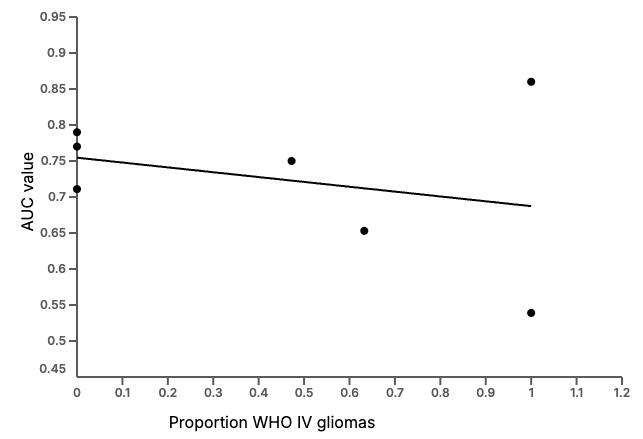


| **Best-fit values** |  |
| --- | --- |
| Slope | -0.06729 ± 0.09767 |
| Y-intercept | 0.7546 ± 0.05979 |
| X-intercept | 11.21 |
| 1/Slope | -14.86 |
| **95% Confidence Intervals** |  |
| Slope | -0.3184 to 0.1838 |
| Y-intercept | 0.6009 to 0.9083 |
| X-intercept | 2.689 to +inf |
| **Goodness of Fit** |  |
| R Square | 0.08669 |
| Sy.x | 0.1090 |
| **Is slope significantly non-zero?** |  |
| F | 0.4746 |
| DFn,DFd | 1,5 |
| P Value | 0.5215 |
| Deviation from horizontal? | Not significant |

### IDH prediction by studies using rADCmin ROI measurements.


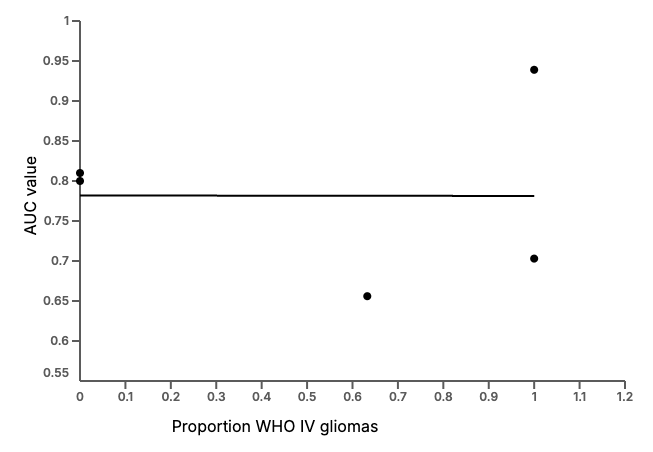


| **Best-fit values** |  |
| --- | --- |
| Slope | -0.0006368 ± 0.1254 |
| Y-intercept | 0.7819 ± 0.08691 |
| X-intercept | 1228 |
| 1/Slope | -1570 |
| **95% Confidence Intervals** |  |
| Slope | -0.3999 to 0.3986 |
| Y-intercept | 0.5053 to 1.059 |
| X-intercept | 2.429 to +inf |
| **Goodness of Fit** |  |
| R Square | 8.591e-6 |
| Sy.x | 0.1263 |
| **Is slope significantly non-zero?** |  |
| F | 2.577e-5 |
| DFn,DFd | 1,3 |
| P Value | 0.9963 |
| Deviation from horizontal? | Not significant |

# Funnel Plots

### ADCmean (ROI) prediction of IDH.


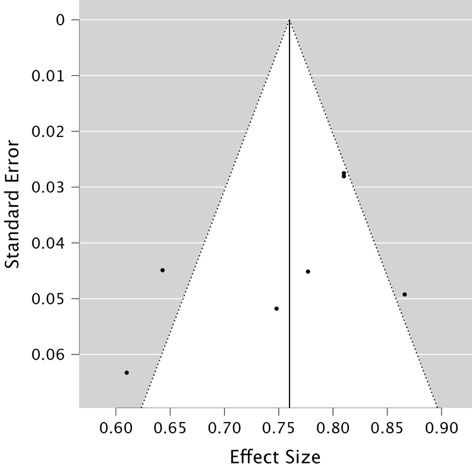


| **Regression test for Funnel plot asymmetry ("Egger's test")** | | | | | |
| --- | --- | --- | --- | --- | --- |
|  | | **z** | | **p** | |
| sei |  | -1.897 |  | 0.058 |  |
|  | | | | | |

### ADCmean (VOI) prediction of IDH


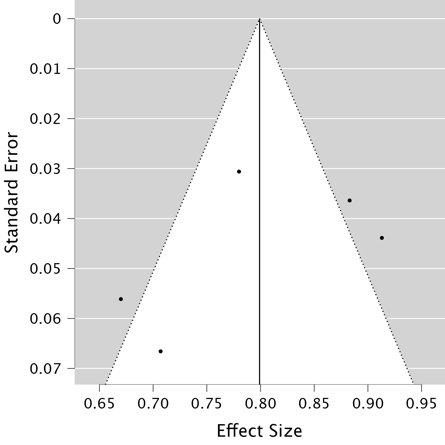


| **Regression test for Funnel plot asymmetry ("Egger's test")** | | | | | |
| --- | --- | --- | --- | --- | --- |
|  | | **z** | | **p** | |
| sei |  | -1.502 |  | 0.133 |  |
|  | | | | | |

### ADCmin (ROI) prediction of IDH


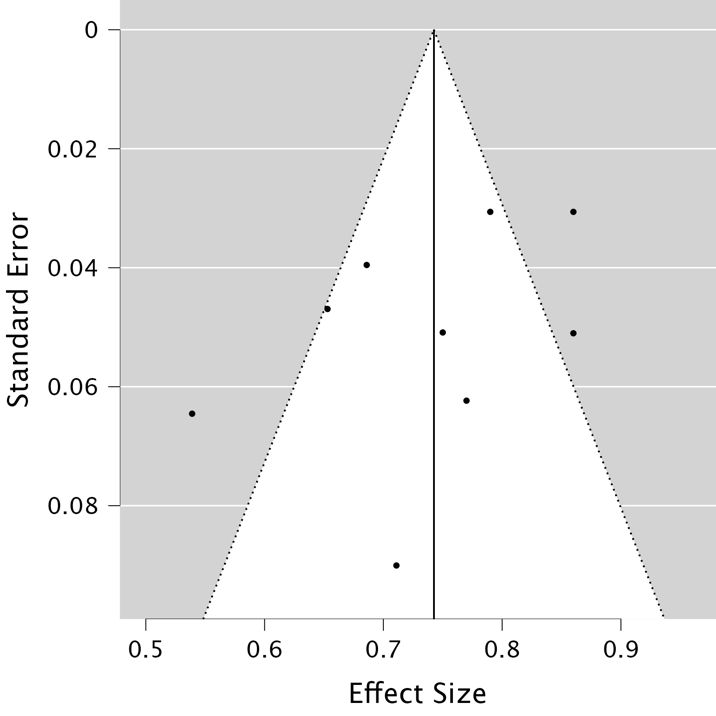


| **Regression test for Funnel plot asymmetry ("Egger's test")** | | | | | |
| --- | --- | --- | --- | --- | --- |
|  | | **z** | | **p** | |
| sei |  | -1.449 |  | 0.147 |  |
|  | | | | | |

### rADCmin (ROI) prediction of IDH


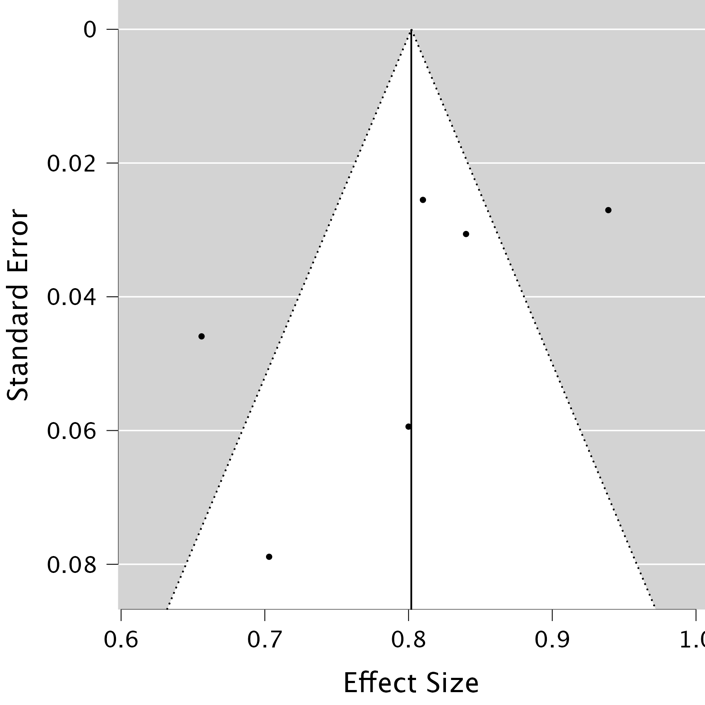


| **Regression test for Funnel plot asymmetry ("Egger's test")** | | | | | |
| --- | --- | --- | --- | --- | --- |
|  | | **z** | | **p** | |
| sei |  | -1.850 |  | 0.064 |  |
|  | | | | | |
